# The cell wall hydrolase Pmp23 is important for assembly and stability of the division ring in *Streptococcus pneumoniae*

**DOI:** 10.1038/s41598-018-25882-y

**Published:** 2018-05-15

**Authors:** Maxime Jacq, Christopher Arthaud, Sylvie Manuse, Chryslène Mercy, Laure Bellard, Katharina Peters, Benoit Gallet, Jennifer Galindo, Thierry Doan, Waldemar Vollmer, Yves V. Brun, Michael S. VanNieuwenhze, Anne Marie Di Guilmi, Thierry Vernet, Christophe Grangeasse, Cecile Morlot

**Affiliations:** 1 Institut de Biologie Structurale (IBS), University Grenoble Alpes, CEA, CNRS, 38000 Grenoble, France; 2 Molecular Microbiology and Structural Biochemistry (MMSB), CNRS, University Lyon 1, UMR 5086, Lyon, 69007 France; 3 Centre for Bacterial Cell Biology, Institute for Cell and Molecular Biosciences, Newcastle University, Newcastle upon Tyne, NE2 4AX UK; 4 Laboratoire de Chimie Bactérienne, Institut de Microbiologie de la Méditerranée, University Aix-Marseille, CNRS, UMR7283, 13009 Marseille, France; 5 Indiana University, Departments of Biology and Chemistry, Bloomington, IN 47405 USA; 6 Present Address: Indiana University, Department of Biology, Bloomington, IN 47405 USA; 7 Present Address: Northeastern University, Department of Biology, Boston, MA 02115 USA

## Abstract

Bacterial division is intimately linked to synthesis and remodeling of the peptidoglycan, a cage-like polymer that surrounds the bacterial cell, providing shape and mechanical resistance. The bacterial division machinery, which is scaffolded by the cytoskeleton protein FtsZ, includes proteins with enzymatic, structural or regulatory functions. These proteins establish a complex network of transient functional and/or physical interactions which preserve cell shape and cell integrity. Cell wall hydrolases required for peptidoglycan remodeling are major contributors to this mechanism. Consistent with this, their deletion or depletion often results in morphological and/or division defects. However, the exact function of most of them remains elusive. In this work, we show that the putative lysozyme activity of the cell wall hydrolase Pmp23 is important for proper morphology and cell division in the opportunistic human pathogen *Streptococcus pneumoniae*. Our data indicate that active Pmp23 is required for proper localization of the Z-ring and the FtsZ-positioning protein MapZ. In addition, Pmp23 localizes to the division site and interacts directly with the essential peptidoglycan synthase PBP2x. Altogether, our data reveal a new regulatory function for peptidoglycan hydrolases.

## Introduction

Bacterial division results from the combination of membrane constriction with expansion and remodeling of the cell wall. The main constituent of the cell wall is the peptidoglycan (PG), a stress-bearing mesh made of glycan chains crosslinked by peptides^1^. PG expansion and remodeling requires three major classes of enzymes: the SEDS (Shape, elongation, division and sporulation) polymerases RodA and FtsW, which elongate the glycan chains, the penicillin-binding proteins (PBPs), which catalyze the polymerization of the glycan chains and the crosslinkage of the peptides, and the cell wall hydrolases (CWHs), which carry various cell wall degrading and remodeling activities^2–5^. The enzymatic activity of many CWHs is known but for most of them, their function in bacterial morphogenesis and division remains unclear. Known CWH functions include regulation of PG precursor availability, PG maturation, septum splitting, cell separation and autolysis^4,5^. It has also been proposed that some CWHs cooperate with synthases to cleave within the existing PG polymer to allow insertion of new material, but direct evidence for such mechanism is still lacking^6^. While the role of the CWHs involved in cell separation and autolysis has been rather well studied, little is known regarding the cellular function of the other ones, especially those involved in PG maturation, which includes the regulation of the length of the glycan and peptide chains, as well as the degree of crosslinking.

During the bacterial division cycle, cell wall synthesis and remodeling are coordinated with membrane constriction, led by the tubulin homologue FtsZ. This coordination relies on a complex network of transient functional and physical interactions, yet to be fully identified and characterized, between SEDS proteins, PBPs, CWHs, FtsZ and other proteins of the divisome with structural or regulatory functions. The tubulin-homologue FtsZ, which polymerizes at midcell into a ring-like structure called the Z-ring^7^, has a major role in this coordination mechanism since it recruits directly or indirectly various division proteins to the division site, including some of the PBPs and CWHs^8–11^. Assembly and stability of the Z-ring is thus essential for proper division. In the opportunistic human pathogen *Streptococcus pneumoniae*, the MapZ protein (also named LocZ) regulates Z-ring positioning, orientation, stability and constriction at midcell through direct interaction with FtsZ^12–14^. In addition, MapZ is a substrate of the Ser/Thr kinase StkP and both phosphorylated and non-phosphorylated forms of MapZ are required for proper Z-ring formation and dynamics^12^. Other pneumococcal proteins with a key regulatory function in cell division are CWHs since impairment of their activity or localization leads to severe shape and division defects^15–20^. However, their precise regulatory function in these processes remains unclear. Investigating this question in model organisms like *Escherichia coli* and *Bacillus subtilis* is not an easy task because these bacteria possess several tens of CWHs with redundant functions^4,5^. By contrast, the ovococcus *S*. *pneumoniae* does not only possess a small number of CWHs (12 pneumococcal CWHs have been identified so far), but they also appear to have non-redundant functions since single deletions are sufficient to observe shape and division defects^15–20^. Among the CWHs involved in pneumococcal growth and division, the *N*-acetylmuramoyl-L-alanine amidase/endopeptidase PcsB is required for septum splitting^17,21^, the putative lytic transglycosylase MltG is required for peripheral cell wall synthesis^20^, the D,D-carboxypeptidase PBP3 (DacA) and the L,D-carboxypeptidase DacB limit the amount of crosslinking by trimming the pentapeptide substrates of the PBP synthases^16,19,22–24^, and the endo-β-*N*-acetylglucosaminidase LytB is responsible for the physical and final separation of daughter cells^25^. A sixth CWH, called Pmp23 (*spr0930* gene), is involved in *S*. *pneumoniae* cell division and morphogenesis. Pmp23, which carries a glycoside hydrolase domain that is conserved in many Gram-positive bacteria (including various human pathogens), was originally proposed to behave as a putative *N*-acetylmuramidase (lytic transglycosylase or lysozyme) in *S*. *pneumoniae*^18,26^. Its deletion results in enlarged cells and misplaced septa but its exact function in *S*. *pneumoniae* morphogenesis and division has remained mysterious so far^18,19,27^. In this work, we investigated the role of Pmp23 in the localization and activity of *S*. *pneumoniae* division and cell wall synthesis machinery. Using 3D homology modeling, genetics, fluorescence microscopy and protein-protein interaction experiments, we provide data supporting the idea that Pmp23 is a bacterial lysozyme involved in the stability of the division machinery, revealing a new connection between cell wall metabolism and cell division.

## Results

### Pmp23 displays homology with bacterial lysozymes

**Figure 1:**
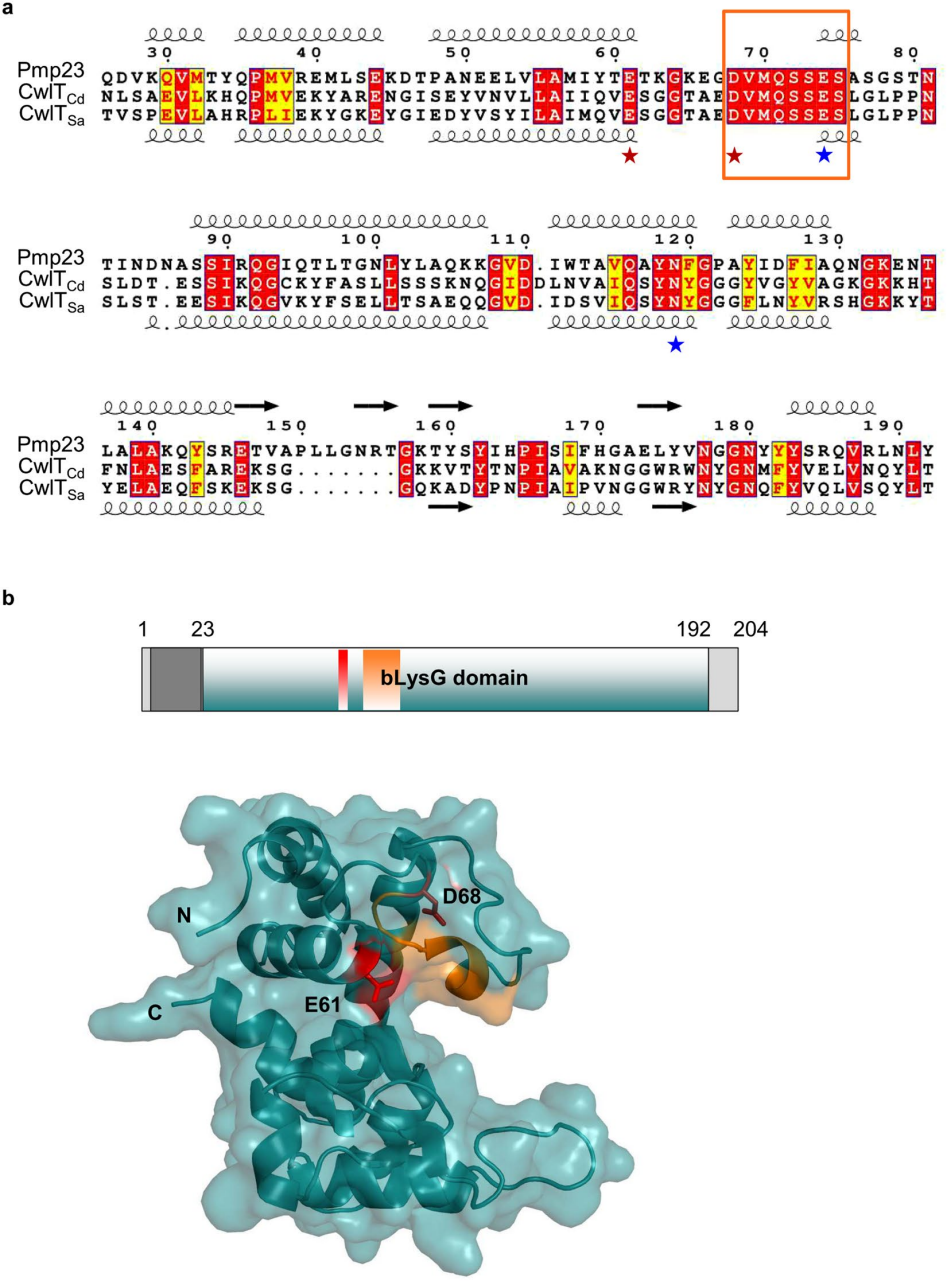
Pmp23 displays homology to bacterial lysozymes. (**a**) The sequence of Pmp23 from *S. pneumoniae* R6 was aligned with CwlT from *C. difficile* 630 (CwlT_Cd_) and *S. aureus* Mu50 (CwlT_Sa_). Conserved residues are shown in red boxes, similar residues in yellow boxes with red characters. The secondary structures of CwlT_Sa_ (PDB code 4FDY) and the predicted ones for Pmp23 are indicated below and above the sequence alignment, respectively. Residues are numbered according to Pmp23. The catalytic Glu and Asp residues of the bLysG domains are highlighted with red stars, the lysozyme-specific Asn and Glu with blue stars and the bLysG-specific DVMQSSES motif with an orange box. Note that Pmp23 possesses all these residues (E61, D68, E74, N119). (**b**) Upper panel: topology of Pmp23 showing the transmembrane segment (in dark grey) and the extracellular bLysG domain (in deepteal). Lower panel: ribbon and surface representation of the Pmp23 model based on the structure of CwlT from *C. difficile* (PDB code 4HPE). The N- and C-termini are labeled. The E61 and D68 catalytic residues are shown in red and the DVMQSSES motif is colored in orange.

In a previous work, Pmp23 was proposed to belong to the *N*-acetylmuramidase family^18,26^, which includes lytic transglycosylases (LTs) and goose(G)-type lysozymes. Lysozymes and LTs are both sugar hydrolases that cleave the β-1,4 Mur*N*Ac-Glc*N*Ac glycosidic bond (*N*-acetylmuramidase activity), but LTs use the C-6 hydroxyl of Mur*N*Ac instead of water as a nucleophile in the cleavage reaction, generating a product ending with 1,6-anhydro Mur*N*Ac. The enzymatic activity of G-type lysozymes and LTs all require a Glu residue that is conserved in Pmp23 (E61) but near this catalytic position, G-type lysozymes carry an Asn and a second negatively charged catalytic residue that are not conserved in LTs^28,29^ and are present in Pmp23 (N119 and E74), suggesting that Pmp23 might be a G-type lysozyme (Fig. S1). However, the low sequence identity of Pmp23 with the G-type lysozyme (10% over 186 residues) prompted us to revisit this analysis. A search for structural homologues in the Protein Data Bank through the NCBI server (http://blast.ncbi.nlm.nih.gov/) identified the bacterial lysozyme domain (bLysG) of CwlT from *Clostridium difficile* (Cwlt_Cd_) and *Staphylococcus aureus* (Cwlt_Sa_), which display 32% and 29% identity with Pmp23, respectively (Fig. 1a). We then performed 3D homology modeling through the Swiss Model server (http://swissmodel.expasy.org/), using the bLysG domain of Cwlt_Cd_ (PDB code 4HPE) and Cwlt_Sa_ (PDB code 4FDY) as templates^29^ (Figs 1b and S1b,c). Both Pmp23_4HPE_ and Pmp23_4FDY_ models were predicted with high-confidence factors (mean value ± SD, 0.7 ± 0.1 for Pmp23_4HPE_ and Pmp23_4FDY_) and most exclusively contain α-helices that form two N- and C-terminal lobes delimiting the putative active site groove, which traverses one face of the protein (Figs 1b and S1b,c). Within the active site, the catalytic Glu and Asp residues of bLysG domains^28^ (E81 and D88 in CwlT_Cd_, E83 and D90 in CwlT_Sa_) are conserved in Pmp23 and correspond to positions E61 and D68 (Fig. 1). In addition, the DVMQSSES sequence motif, which is conserved in bLysG domains but absent in LTs and G-type lysozymes and defines the bLysG family^28,29^, is strictly conserved in Pmp23 (D_68_VMQSSES) (Figs 1 and S1). Pmp23 thus possesses all the specific features of a bacterial lysozyme.

### Deletion of *pmp23* and inactivation of its predicted bacterial lysozyme activity cause morphological defects

**Figure 2:**
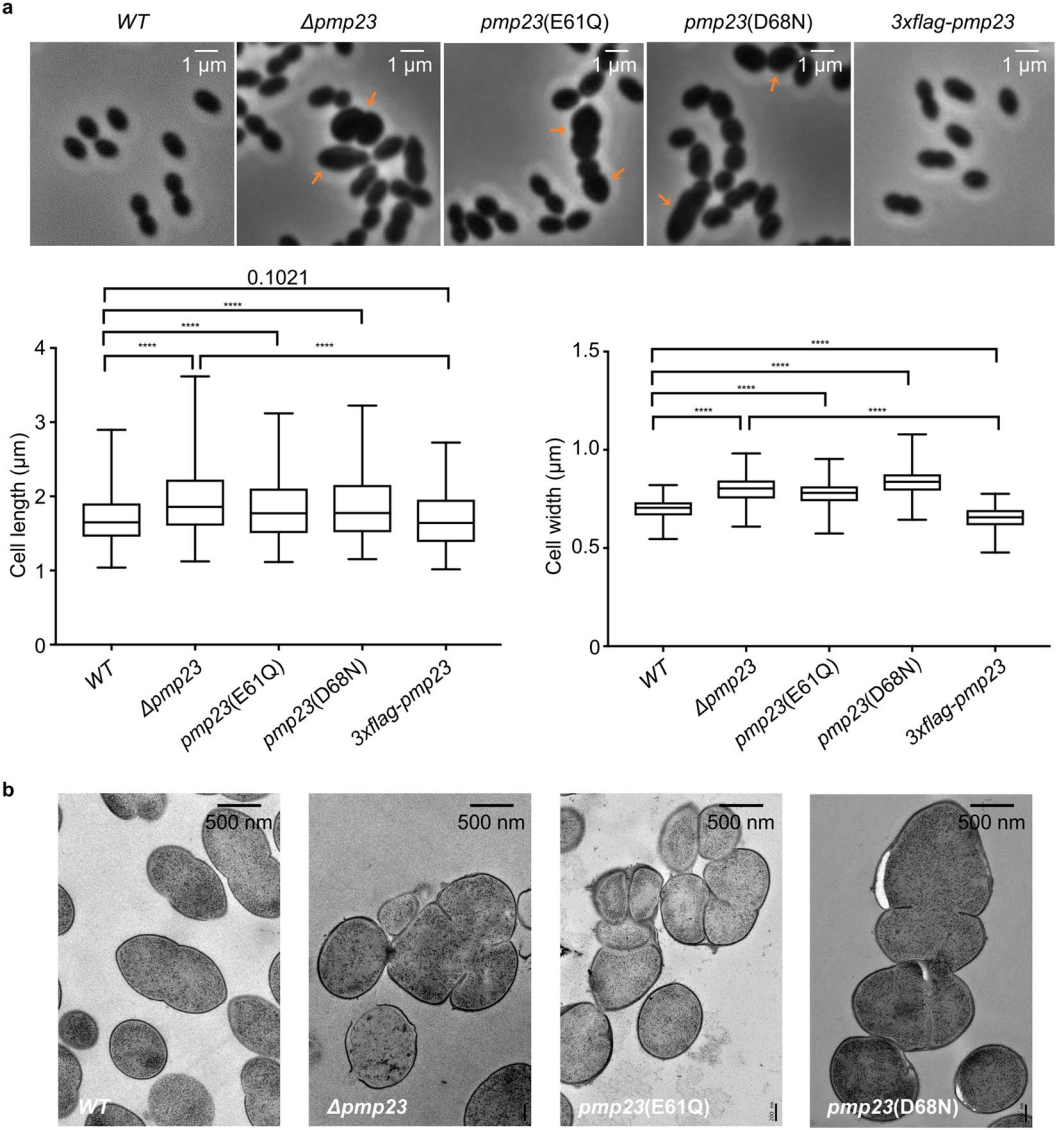
Characterization of wild-type and *pmp23* mutant *S. pneumoniae* cell morphology. (**a**) Phase contrast images of exponentially growing wild-type (*WT*), *Δpmp23*, *pmp23*(E61Q), *pmp23*(D68N) and *Δpmp23-3xflag-pmp23* (*3xflag-pmp23*) *S. pneumoniae* cells. Orange arrows indicate cells with a distorted shape and/or division defects. Distributions of cell length and width among the cell population in wild-type (n = 370), *Δpmp23* (n = 643), *pmp23*(E61Q) (n = 740), *pmp23*(D68N) (n = 599) and *Δpmp23-3xflag-pmp23* (n = 590) strains are represented with box plots showing the interquartile range (25^th^ and 75^th^ percentile), the median value and whiskers for minimum and maximum values. P-values from the U test of Mann-Whitney are indicated with an exact number when no significant difference is observed (p-value > 0.05) or with an asterisk when the observed differences are statistically significant (p-value < 0.05). Quadruple asterisks indicate p-values < 0.0001. (**b**) Electron microscopy images of thin sections of wild-type, *Δpmp23*, *pmp23*(E61Q) and *pmp23*(D68N) *S. pneumoniae* cells.

Deletion of the *pmp23* gene using an antibiotic insertion cassette was previously shown to affect cell morphogenesis and division in *S*. *pneumoniae* R6 or D39^18,19,27^. To verify that the phenotypes of these strains were not due to a polar effect on the expression of neighboring genes, we constructed a markerless *pmp23* deletion strain of *S*. *pneumoniae* R6 (*Δpmp23*). Median values of 1.65 ± 0.22 µm (n = 370) and 1.86 ± 0.29 µm (n = 643) were obtained for the cell length of wild-type and *Δpmp23* cells, respectively (Fig. 2a). Median values of 0.70 ± 0.03 µm and 0.80 ± 0.05 µm were obtained for the cell width of wild-type and *Δpmp23* cells, respectively. Statistical analyses of the length and width distributions indicated that the observed differences between the two strains are significant (U test of Mann-Whitney, p-values <0.0001 for all analyses, see Fig. 2a). In the absence of Pmp23, cells are thus slightly but significantly longer and larger than those of the wild-type strain. In addition, ~13% of *Δpmp23* cells had a distorted shape and/or division defects. Analysis of these abnormal cells by electron microscopy revealed the presence of misplaced, aborted and sometimes multiple septa (Figs 2b and S2c). When the *pmp23* deletion strain was complemented with a *3xflag-pmp23* construct, normal cell morphology was almost completely restored, with only 1% of cells being misshaped (median cell length value of 1.64 ± 0.27 µm, p-value = 0.1021; median cell width value of 0.66 ± 0.04 µm, p-value < 0.0001, when compared with the wild-type strain), suggesting that the shape and division defects of the *Δpmp23* cells are likely due to the absence of Pmp23 (Fig. 2a).

To reconstitute Pmp23 hydrolytic activity *in vitro*, full-length Pmp23 fused to the Glutathione S-Transferase (GST-Pmp23) was purified and solubilized with the non-ionic detergent DDM, and incubated overnight at 37 °C in the presence of purified sacculi from wild-type and *Δpmp23* pneumococcal strains (see the *Methods* section). The use of sacculi from the *Δpmp23* strain was meant to increase the proportion of specific Pmp23 substrate in the purified sacculi. Despite intensive efforts to provide a suitable substrate for Pmp23 (removal of teichoic acids, amidase digestion for removal of peptide chains, and chemical *N*-acetylation), we were unable to detect peptidoglycan degradation products. Accordingly, we sought to determine whether the predicted catalytic residues are important for Pmp23 function *in vivo* and assess the importance of its putative lysozyme activity for pneumococcal division and morphogenesis.

When mutations E61Q and D68N were introduced in the endogenous *pmp23* gene, the mutant cells displayed shape defects similar to those observed when *pmp23* was deleted (Figs 2 and S2d,e). Indeed, *pmp23*(E61Q) and *pmp23*(D68N) cells were longer than wild-type cells, with median cell length values of 1.77 ± 0.29 µm (n = 740) and 1.77 ± 0.31 µm (n = 599), respectively. The mutant cells were also larger, with median cell width values of 0.78 ± 0.04 µm for strain *pmp23*(E61Q) and 0.84 ± 0.04 µm for strain *pmp23*(D68N) (Fig. 2a). Statistical analyses of the length and width distributions indicated that the observed differences between the wild-type strain and these two *pmp23* mutant strains are significant (Mann-Whitney p-values < 0.0001, see Fig. 2a). Finally, ~16% of mutant cells showed division defects (Figs 2b and S2d,e). Importantly, the stability of the mutant proteins and the wild-type control were similar (Fig. S2a).

In conclusion, the overall phenotype of the *Δpmp23* strain suggests that Pmp23 is involved in pneumococcal division and maintenance of cell shape. In addition, the fact that mutations E61Q and D68N phenocopy the *pmp23* deletion supports the idea that Pmp23 is an active lysozyme *in vivo* and that this enzymatic activity is required for proper morphogenesis and division of *S*. *pneumoniae*.

### Inactivation of Pmp23 leads to aberrant localization of FtsZ and the cell wall synthesis machinery

**Figure 3:**
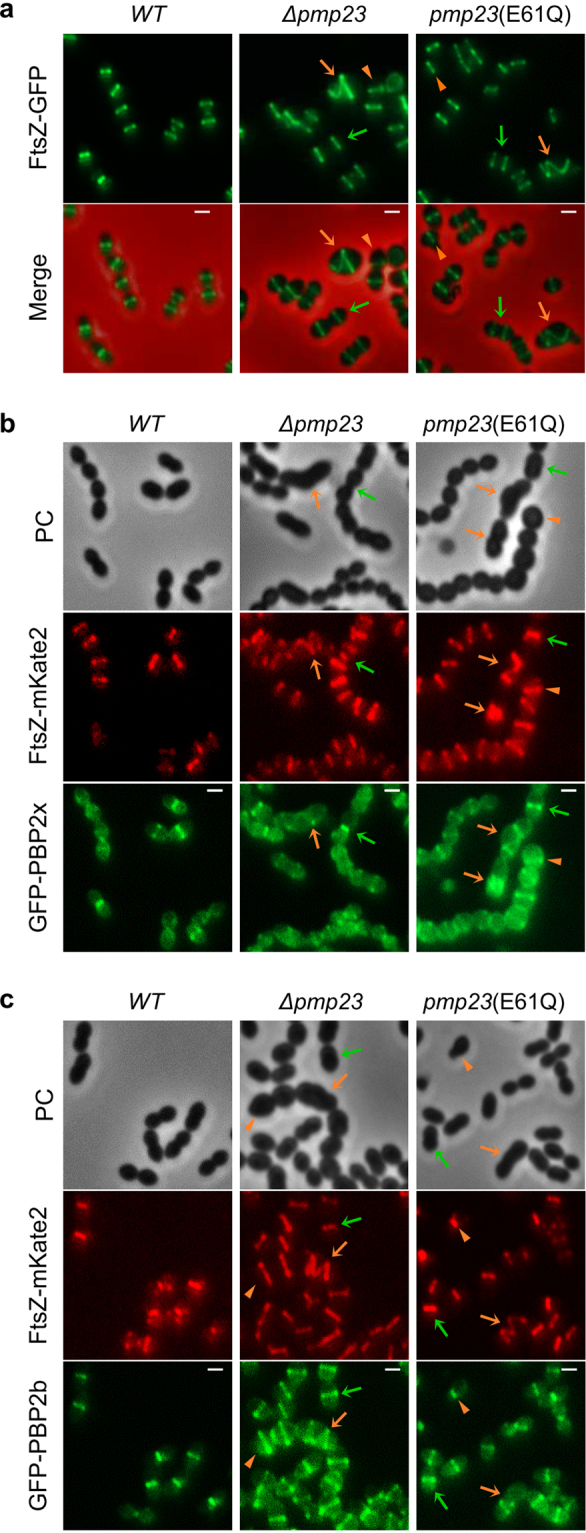
FtsZ and PBP localization in wild-type and *pmp23* mutant cells. (**a**) Live *S. pneumoniae* cells expressing an endogenous copy of *ftsZ-gfp* in wild-type (WT), *Δpmp23* and *pmp23*(E61Q) genetic backgrounds. Examples of cells with wild-type FtsZ localization (green arrows) and distorted cells with asymmetric (orange arrowheads) or helical (orange arrows) FtsZ localization are indicated. GFP fluorescence and merged images (between the GFP and the phase contrast channels) are shown. Scale bars = 1 μm. (**b**,**c**). Live *S. pneumoniae* cells expressing the endogenous copy of *ftsZ-gfp* together with either *gfp-pbp2x* (**b**) or *gfp-pbp2b* (**c**) in wild-type (WT), *Δpmp23* and *pmp23*(E61Q) genetic backgrounds. Examples of cells with wild-type FtsZ and PBP localization (green arrows) and distorted cells with asymmetric (orange arrowheads) or helical (orange arrows) FtsZ and PBP localization are indicated. Phase contrast (PC), GFP and mKate2 fluorescence images are shown. Scale bars = 1 μm.

The morphological and division defects observed with the *pmp23* mutant strains suggest that the localization or activity of the cell wall synthesis machinery is perturbed when Pmp23 is absent or inactive. To test this idea, we first investigated the localization of the major division protein FtsZ, which scaffolds the cell wall synthesis machinery, and the cell wall synthases PBP2x and PBP2b in wild-type, *Δpmp23* and *pmp23*(E61Q) strains. To do so, we expressed previously described *ftsZ-gfp*, *ftsZ-mkate2*, *gfp-pbp2x* and *gfp-pbp2b* fusions at their endogenous locus, under the control of their native promoter^11^. Importantly, production of these fluorescent fusion proteins did not modify the morphology of the wild-type and the Pmp23 mutant strains. Most *Δpmp23* and *pmp23*(E61Q) cells (88%, n = 310 and 85%, n = 232, respectively), including those with slightly enlarged dimensions, displayed a wild-type localization of FtsZ, PBP2x and PBP2b, with midcell positioning of ring-like structures, progressive constriction and relocalization at the division sites of the future daughter cells^11,30–32^ (Figs 3 and S3a). However and importantly, all cells with major morphological defects (12% of *Δpmp23* cells; 15% of *pmp23*(E61Q) cells) showed aberrant FtsZ, PBP2x and PBP2b localizations, including annular structures positioned asymmetrically and helix-like structures (Figs 3 and S3a). Aberrant FtsZ structures always co-localized with PBP2x and PBP2b (Fig. 3b,c). Time-lapse experiments performed on *pmp23*(E61Q) cells expressing the *ftsZ-gfp* fusion further showed that asymmetric and helical FtsZ structures can form in cells with or without primary morphological defects (Fig. S4). In addition, helices can either form as soon as FtsZ becomes visible in the daughter cell or arise from single Z-rings that fall apart at some point (Fig. S4). Finally, asymmetric Z-rings and more unexpectedly some of the helical Z-rings are able to constrict, leading to the generation of daughter cells with unequal size (Fig. S4). We verified that FtsZ-GFP levels are not affected in the absence of Pmp23 or in the presence of the Pmp23(E61Q) variant (Fig. S3b), and that helical FtsZ structures were also observed when the native copy of FtsZ was immunolabeled in *Δpmp23* and *pmp23*(E61Q) cells (Fig. S3c). Altogether, these observations suggest that in the absence of Pmp23 or in the presence of an inactive variant, the division and cell wall synthesis machinery is most of the time able to assemble at midcell, but in some cells degenerates into helical structures. Furthermore, helical or asymmetric Z-rings can also form at the onset of the cell cycle and none of these structures constitute dead ends for the division process.

### Cell wall synthesis is not affected by Pmp23 inactivation

**Figure 4:**
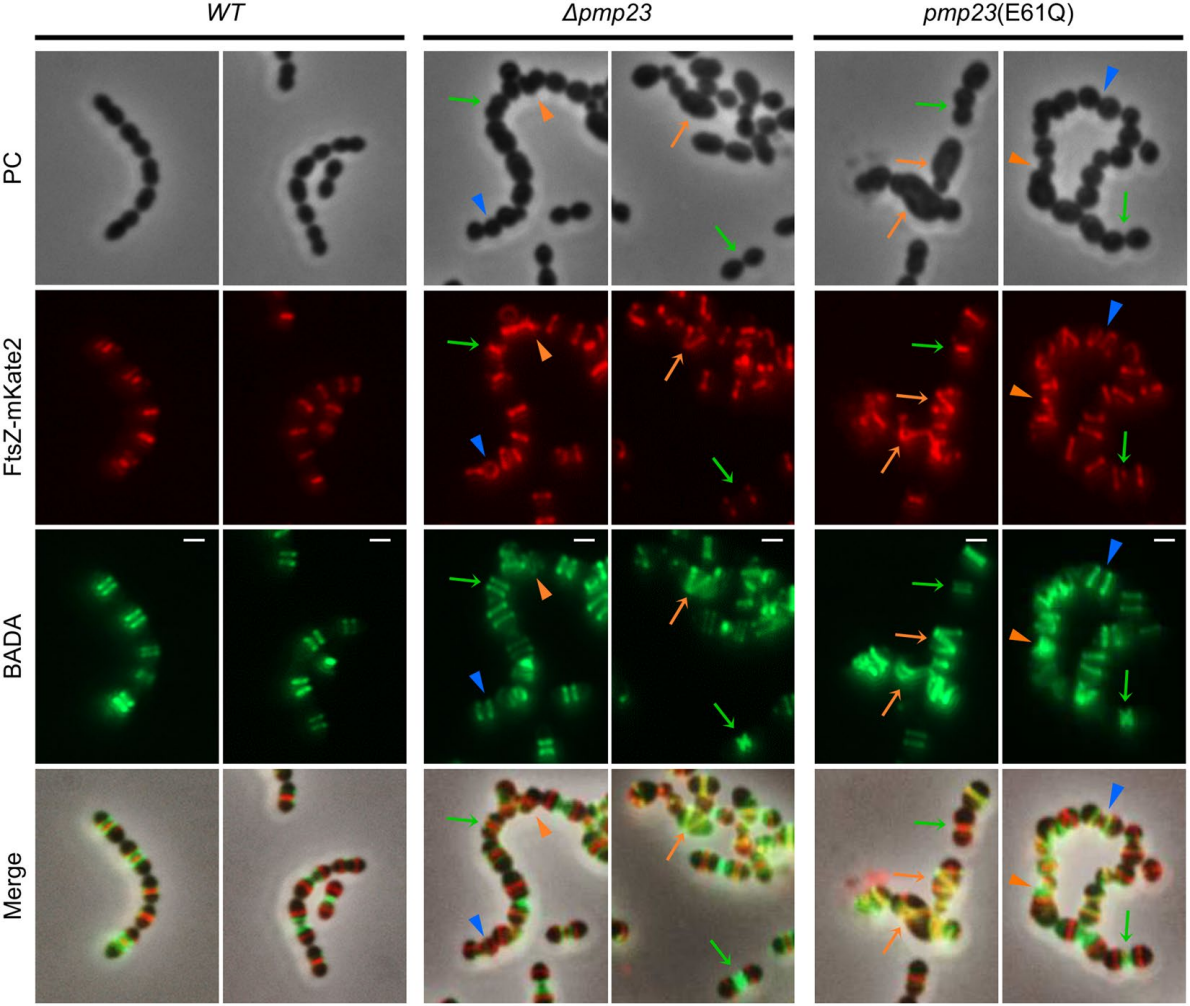
FtsZ localization and PG labeling in wild-type and *pmp23* mutant cells. Pulse-chase labeling of PG with BADA in live *S. pneumoniae* cells expressing an endogenous *ftsZ-mkate2* fusion in wild-type (*WT*), *Δpmp23* and *pmp23*(E61Q) genetic backgrounds. Examples of cells with wild-type FtsZ localization and PG labeling (green arrows), distorted cells with asymmetric (orange arrowheads) or helical (orange arrows) FtsZ localization and PG labeling are indicated. Blue arrowheads point at cells in which the Z-ring and PG labeling are oriented in different planes. Phase contrast (PC), mKate2 and BADA fluorescence, and merged images are shown. Scale bars = 1 μm.

We next wondered whether the aberrant FtsZ, PBP2x and PBP2b structures observed in *Δpmp23* and *pmp23*(E61Q) cells might arise from altered cell wall synthesis. To test this idea, we monitored cell wall synthesis activity by pulse-chase labeling of new PG using fluorescent D-amino acids (FDAAs)^33^. Wild-type, *Δpmp23* and *pmp23*(E61Q) cells expressing an FtsZ-mKate2 fusion were grown shortly in the presence of the BADA label, then the excess dye was washed away and cells were further incubated for 4 min. Most mutant cells (74%, n = 206 for *Δpmp23*; 70%, n = 131 for *pmp23*(E61Q)) displayed a wild-type PG labeling, which appeared as a single or double bands flanking the Z-ring depending on the advancement of the cell cycle (Fig. 4). In *Δpmp23* and *pmp23*(E61Q) cells displaying major morphological defects (21% and 25%, respectively), PG labeling still co-localized with or flanked FtsZ although FtsZ displayed asymmetric or helical structures (Fig. 4). Given that FtsZ always co-localizes with PBPs^11^ (Fig. 3b,c), these observations suggest that in the absence of Pmp23 or in the presence of an inactive enzyme, the PG synthesis machinery remains active even though it displays abnormal localization. Very surprisingly in a small proportion of *Δpmp23* and *pmp23*(E61Q) cells with no major morphological defects (5% for both strains), the Z-ring and PG labeling were oriented in different planes, indicating that the Z-ring had changed orientation during the chase period (Fig. 4). This unexpected observation further suggests that Pmp23 inactivation causes instability of the orientation of the division and PG synthesis machinery.

### MapZ localization is altered in *pmp23* mutant cells

**Figure 5:**
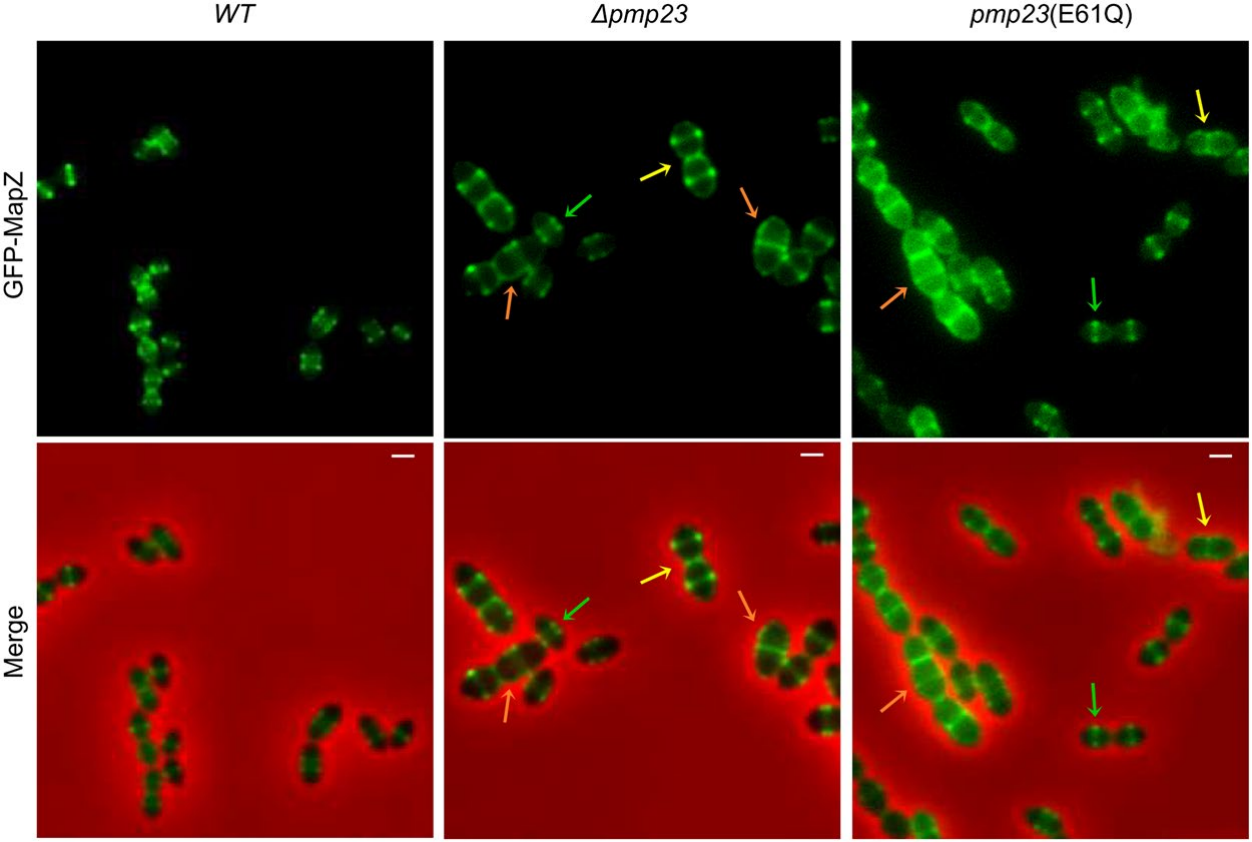
MapZ localization in wild-type and *pmp23* mutant cells. Live *S. pneumoniae* cells expressing an endogenous copy of *gfp-mapZ* in wild-type (*WT*), *Δpmp23* and *pmp23*(E61Q) genetic backgrounds. Examples of cells displaying wild-type morphology and wild-type MapZ localizations (green arrows), enlarged cells with a slight membrane delocalization of MapZ (yellow arrows) and abnormal morphology with aberrant MapZ localizations (orange arrows). GFP fluorescence and merged images (between the GFP and the phase contrast channels) are shown. Scale bars = 1 μm.

Because cell wall synthesis is still active when Pmp23 is inactive, we wondered whether aberrant FtsZ and PBP structures might arise from impaired localization of MapZ, which is involved in FtsZ positioning, orientation stability and constriction^12–14^. To investigate this, we expressed a previously described *gfp-mapZ* fusion^12^ in wild-type, *Δpmp23* and *pmp23*(E61Q) cells. Wild-type cells displayed the previously described MapZ localization profile: assembly of a single septal ring at the onset of the cell cycle, splitting of this initial ring and progressive separation of the double ring, appearance and constriction of a third ring at midcell (Figs 5 and S5a). While *Δpmp23* and *pmp23*(E61Q) cells with wild-type morphology and dimensions displayed typical MapZ localization profiles, cells with enlarged cell dimensions (14% (n = 370) of *Δpmp23* cells and 17% (n = 361) of *pmp23*(E61Q) cells) displayed a slight delocalization of MapZ around the cell periphery (Figs 5 and S5a). In addition, all *Δpmp23* and *pmp23*(E61Q) cells with major morphological defects (13% of *Δpmp23* cells and 15% of *pmp23*(E61Q) cells) showed aberrant MapZ structures, including y-shaped, aggregated structures, membrane delocalization or asymmetric positioning of MapZ rings relative to the division site (Figs 5 and S5a). GFP-MapZ levels were not affected by *pmp23* deletion or expression of the inactive variant (Fig. S5b). We thus conclude that Pmp23 is not required for the assembly of the MapZ rings but Pmp23 inactivation leads to partial mislocalization of MapZ.

We next sought to investigate why MapZ localization is affected when Pmp23 is inactive. MapZ localization was previously shown to be driven by its C-terminal PG binding domain^34^. We purified sacculi from wild-type and *Δpmp23* cells, and analyzed the PG composition (see the *Methods* section). The glycan strands in pneumococcal PG are very long, most of them containing more than 50 disaccharides^35,36^. The purified sacculi thus had to be treated with mutanolysin before analysis, preventing determination of the average length of the glycan strands. Nevertheless, the analysis of PG composition revealed that the strain lacking *pmp23* had 8% more dimers than monomers, while the wild-type strain had nearly identical proportion of monomers and dimers (Fig. S6). Despite this change in PG composition, MapZ ability to bind the polymer was not significantly affected in pull-down experiments using a recombinant construct of MapZ extracellular domain and purified sacculi from wild-type and *Δpmp23* cells (Fig. S7a). In addition, MapZ phosphorylation did not display significant variation in the *Δpmp23* strain when compared to the wild-type strain (Fig. S7b). Consistent with this observation, the StkP kinase retained septal localization in the *Δpmp23* strain (Fig. S7c).

### Pmp23 is enriched at midcell and interacts with PBP2x

**Figure 6:**
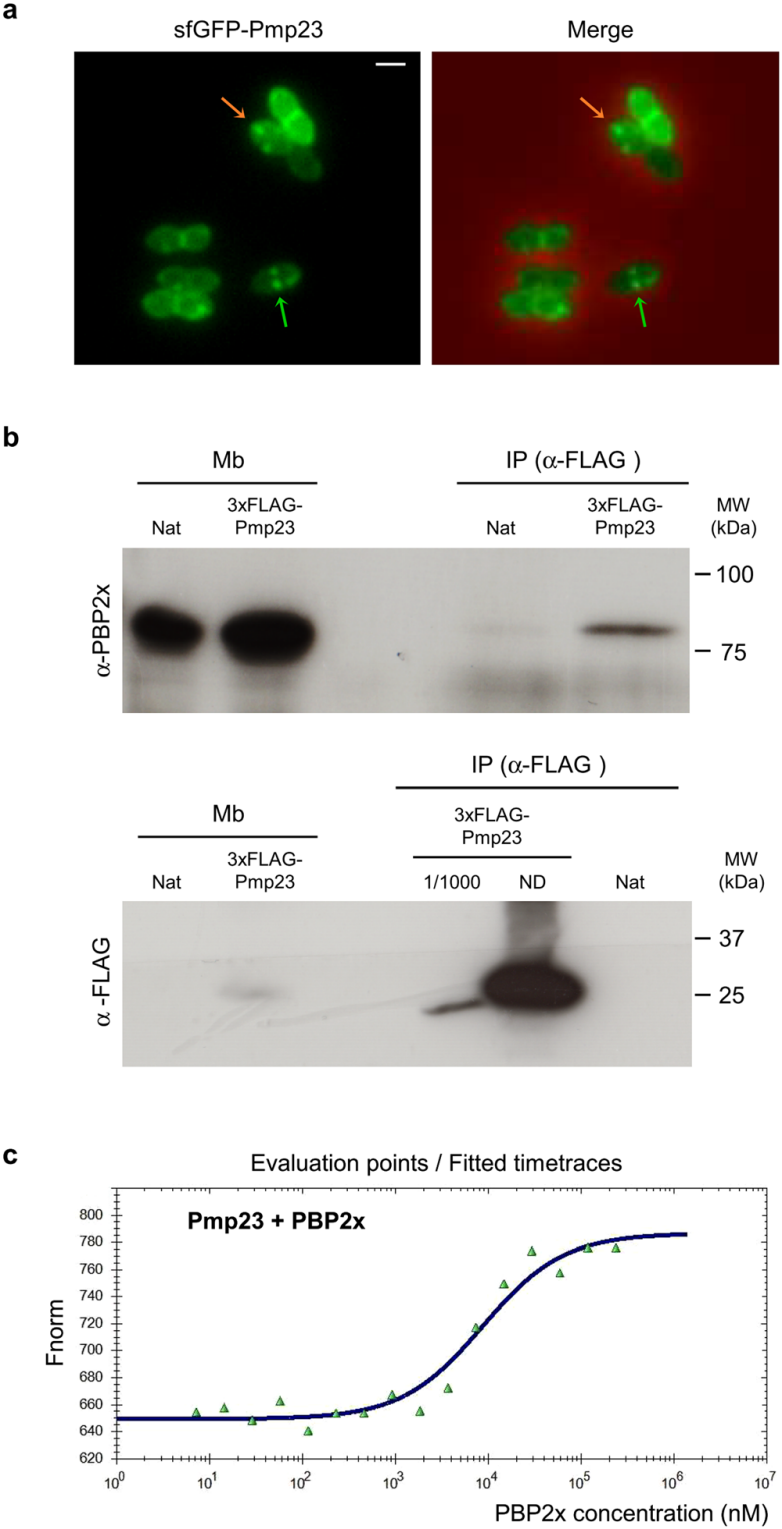
Pmp23 localizes to the division site and interacts with PBP2x. (**a**) Phase contrast (PC) and fluorescence microcopy images of live *S. pneumoniae* cells expressing an ectopic copy of *sfgfp-pmp23*. Examples of cells displaying sfGFP-Pmp23 septal localization (green arrows) and foci (orange arrows) are indicated. GFP fluorescence and merged images (between the GFP and the phase contrast channels) are shown. Scale bars = 1 μm. (**b**) Membrane proteins from S. *pneumoniae* cells expressing the native copy of *pmp23* (Nat) or a *3xflag-pmp23* fusion were solubilized with Triton X-100 and used for immunoprecipitation experiments using anti-FLAG resin. The analyzed samples correspond to the solubilized membrane proteins (Mb) and the immunoprecipitated fractions (IP). The anti-PBP2x serum and anti-FLAG antibodies used for the Western blot detection and molecular weight markers (MW) are indicated. ND, not diluted. (**c**) Affinity measurements by microscale thermophoresis of labeled GST-Pmp23 binding to increasing concentrations of PBP2x. Fnorm (normalized fluorescence = fluorescence after thermophoresis/initial fluorescence) is plotted against PBP2x concentration. Measures are represented by green dots and the fitted curve by a blue line.

Because Pmp23 is predicted to have a glycosidase activity and not a peptidase activity, we hypothesized that the increase in dimer proportion versus monomers in the *pmp23* deletion strain could reflect an aberrant functioning of some peptidoglycan transpeptidases (PBPs), suggesting a potential functional or physical interaction between PBPs and Pmp23. Interestingly, fluorescence microscopy imaging of *S*. *pneumoniae* cells expressing an ectopic *sfgfp-pmp23* fusion revealed that 75% of the cell population (n = 210) displayed a diffuse cytoplasmic and membrane fluorescence signal while 25% of cells showed enriched fluorescence at the septum (Fig. 6a). Diffuse fluorescence might be due to the fact that about 30% of the sfGFP-Pmp23 fusion protein is cleaved, as observed by the presence of a band corresponding to the sfGFP (~30 kDa) in the anti-GFP Western blot shown in Figure S2a. In addition, about 30% of cells displayed fluorescence foci, possibly reflecting the presence of sfGFP-Pmp23 aggregates. These observations thus indicate that the sfGFP-Pmp23 fusion protein is not fully functional. Nevertheless, the observation of septal fluorescence suggests that in 25% of cells, Pmp23 is ideally located to interact with the PBPs. This observation prompted us to perform immunoprecipitation experiments to identify potential PBP partners of Pmp23. To do so, we used anti-FLAG IgGs on detergent-solubilized membrane fractions from pneumococcal cells expressing the *3xflag-pmp23* fusion. Immunoprecipitated fractions were analyzed by western blot using anti-FLAG IgGs and antibodies specific for each of the five high-molecular weight pneumococcal PBPs (PBP2x, PBP2b, PBP1a, PBP1b and PBP2a)^30^. Consistent with the idea that Pmp23 and PBP2x interact in the membranes of *S*. *pneumoniae*, PBP2x was co-immunoprecipitated with 3xFLAG-Pmp23 (Fig. 6b). Importantly, PBP2x was not detected in the immunoprecipitate from an extract derived from a strain that did not express *3xflag-pmp23* (Fig. 6b). To determine whether Pmp23 and PBP2x interact directly *in vitro* and to quantify this interaction, microscale thermophoresis was carried out by titrating the binding of the extracellular domain of PBP2x^31^ to the full-length membrane form of Pmp23. While no binding of Pmp23 was detected with the BSA (bovine serum albumine) control, an affinity constant of 9 ± 0.8 μM was obtained for the Pmp23-PBP2x complex (Fig. 6c). This result indicates that Pmp23 and PBP2x interact directly through their extracellular domain.

## Discussion

This study provides evidence that Pmp23 is neither a LT nor a G-type lysozyme as previously predicted^18,26^ but is most likely a bacterial lysozyme required for proper cell morphogenesis and division. In support of this idea, we show that Pmp23 contains the bLysG-specific DVMQSSES motif and catalytic residues (E61 and D68 in particular), and that mutations E61Q and D68N phenocopy the deletion of the protein.

Indeed when Pmp23 is absent or is mutated on one of the putative catalytic residues E61 or D68, *S*. *pneumoniae* cells are larger and longer, and a portion of them displays severe morphological and division defects. In the large majority of Pmp23 mutant cells with wild-type morphology or minor morphological defects, FtsZ and the PBPs display a wild-type localization pattern, indicating that the division and PG synthesis machinery is most of the time able to assemble at midcell and constrict throughout the cell cycle. However, cells with severe morphological defects all display abnormal FtsZ localizations, including Z-rings assembled at asymmetric positions and helix-like structures. As expected because the Z-ring serves as a scaffolding platform for the cell wall synthesis machinery, PBP2x and PBP2b always co-localize with these abnormal FtsZ structures. In addition, monitoring of PG synthesis showed that PBPs remain active even though FtsZ is mislocalized. Asymmetric or helical FtsZ structures can appear in newborn daughter cells with no primary morphological defects; they can form *de novo* when the FtsZ relocalizes to the division sites of the future daughter cells, or they can arise from regular Z-rings, indicating that in some cells, Pmp23 inactivation impairs the assembly or stability of the Z-ring. In support of this idea, pulse-chase PG labeling experiments revealed that in Pmp23 mutant cells, the Z-ring can change its orientation or structure during the course of the division cycle, while the PBPs are actively synthesizing PG.

Recently, MapZ, a substrate of the Ser/Thr kinase StkP, was shown to locate at the division site before FtsZ and serve as a beacon for FtsZ positioning and orientation at midcell^12–14^. In the absence of MapZ, ~30% of cells display aberrant FtsZ structures^12^. Imaging of MapZ in the absence of Pmp23 or in the presence of an inactive variant revealed that cells with slight dimension defects are always associated with a slight delocalization of MapZ around the cell periphery. Moreover, cells with major morphological defects all display abnormal MapZ structures. These observations suggest that although Pmp23 is not strictly required for MapZ positioning at midcell, its absence leads to partial delocalization of MapZ and sometimes causes aberrant MapZ cellular structures. In addition, the fact that MapZ displays similar localization defects in *Δpmp23* and *pmp23*(E61Q) cells suggests that the role of Pmp23 in MapZ localization is due to its enzymatic activity rather than its physical presence.

Our data are thus consistent with the idea that PG modification by Pmp23 might be important for MapZ localization and stability of its macromolecular structure. Supporting this hypothesis, molecular docking showed that the MapZ extracellular and C-terminal domain could bind the glycan chains of PG, and deletion of this PG-binding domain results in MapZ delocalization^12,34^. For example, MapZ localization might require a certain homogeneity of the glycan chain length or a certain flexibility of the septal PG, and these PG properties might require the lysozyme activity of Pmp23. In the absence of this putative hydrolase, abnormal PG properties due to abnormally long glycan strands could influence the ability of MapZ to interact with PG. However, MapZ binding to PG was not significantly affected when sacculi were purified from a strain deleted for *pmp23* or expressing an inactive variant. We thus favor an alternative hypothesis in which Pmp23 would contribute to a “quality control” function aiming at correcting errors performed by PG synthases in the PG network. In support of this idea, Pmp23 localization is enriched at midcell where all the PG synthases are positioned^11,30,32,37^, it interacts directly with PBP2x and we detected a slightly higher percentage of peptide crosslinks in PG purified from *Δpmp23* mutant cells. Excessively crosslinked glycan strands might not be properly bound by MapZ because of steric hindrance or excessive rigidity of such strands. These improperly incorporated glycan strands might lead to partial delocalization of MapZ unless being degraded by Pmp23. In the absence of Pmp23 housekeeping activity, MapZ delocalization might lead to a subtle delocalization and/or disorientation of FtsZ and the PBPs, first causing slight morphological defects. As the cell cycle proceeds, slight localization and shape defects would be rapidly emphasized by active cell wall synthesis, eventually leading to abnormal structure of the division ring and severe morphological defects. Importantly, this second model is consistent with the previously reported MapZ delocalization upon perturbation of PG synthesis, and MapZ function in Z-ring positioning and orientation^12–14^. Further studies are now needed to determine at the molecular level how MapZ binds PG and how the altered PG composition in the *pmp23* mutants alters MapZ localization.

Many studies support the idea that early division proteins drive the localization and assembly of the cell wall synthesis machinery, which includes PG synthases and PG hydrolases. About 13 years ago, the pneumococcal CWH DacA (PBP3), which carries a D,D-carboxypeptidase activity, was shown to be required for proper orientation of FtsZ and PBP annular structures at midcell^16^. This work seeded the idea that CWHs are important for proper assembly and localization of the division machinery. More recently, similar defects in FtsZ positioning were observed in the absence of DacA (PBP5) and other low-molecular weight PBPs in *E*. *coli*^38^. In the present work, we show that the lysozyme activity of Pmp23 is important for the stability of the earliest structure(s) assembling at the division site in *S*. *pneumoniae*, the MapZ ring(s) that guide Z-ring positioning and orientation at midcell^12–14^. This work thus uncovers a new crosstalk between cell wall metabolism and cell division. Although most CWHs are not essential for bacterial survival in laboratory conditions, they might be important safeguards insuring that bacterial division proceeds properly.

## Methods

### Bacterial strains and plasmids

Strains, plasmids and oligonucleotides used in this study are listed in Tables S1 and S2.

### Growth conditions, media and allelic replacement

*S*. *pneumoniae* strains were grown at 37 °C in CY medium^39^ or in Todd Hewitt medium (TH, BD Sciences). Blood agar plates were made from Columbia agar containing 5% defibrinated horse blood (Difco) and the appropriate antibiotics (2.5 µg/ml tetracycline, 250 µg/ml kanamycin or 200 µg/ml streptomycin). Markerless allelic replacements were performed as described previously^40^ using a two-step procedure based on a bicistronic *kan-rpsL* cassette called Janus to delete the genes of interest, replace them by fluorescent protein fusion or introduce point mutations.

### 3D homology modeling of Pmp23

The Swiss Model server (http://swissmodel.expasy.org/) was used to generate models of the soluble domain of Pmp23 based on the structures of CwlT from *C. difficile* and *S. aureus* (PDB codes 4HPE and 4FDY, respectively)^29^. Both models display high confidence factors (~70%) and global QMEAN scoring functions of about - 4.5.

### Fluorescence microscopy acquisition and analysis

For phase contrast, GFP or mKate2 fluorescence imaging, cells were grown at 37 °C in CY medium until OD_600_ = 0.3, transferred to microscope slides, and observed at room temperature using an Olympus BX61 optical microscope equipped with a UPFLN 100x O-2PH/1.3 objective and a QImaging Retiga-SRV 1394 cooled charge-coupled-device (CCD) camera.

For labeling of cell wall synthesis regions, cells were grown in CY until OD_600_ = 0.3, pelleted at room temperature for 5 min at 4,000 × g, resuspended and incubated for 4 min in CY medium supplemented with 0.1 mM BADA. Cells were finally washed twice with CY medium to discard unbound dye, resuspended into pre-warmed medium and incubated for 4 min at 37 °C prior to microcopy observation.

For immunofluorescence microscopy, cells were grown in CY medium until OD_600_ = 0.3 and fixed for 15 min at room temperature and 45 min on ice in 4% (wt/vol) paraformaldehyde. After three washes in PBS, cells were resuspended in GTE buffer (50 mM glucose, 10 mM EDTA, 20 mM Tris pH 7.5) and transferred onto poly-L-lysine-coated slides. The slides were incubated in PBS-0.2% Triton X-100 (vol/vol) for 5 min, air dried, incubated in methanol at −20 °C for 5 min and air dried again. After rehydration with PBS, the slides were blocked for 60 min at room temperature with PBS-0.2% Triton X-100-5% (wt/vol) BSA (PBS-Triton-BSA). The slides were incubated for 1 h at room temperature with 1:200 of mouse anti-FtsZ antibodies^30^, in PBS-Triton-BSA. The slides were washed with PBS-Triton-BSA and incubated for 1 h with a 1:200 dilution of Cy2-coupled anti-mouse IgG in PBS-Triton-BSA. Following washing, slides were mounted with 5% (wt/vol) polyvinyl alcohol.

Images were acquired using the Volocity software package. Image analysis was performed using the MicrobeTracker Matlab software^41^. Distributions of cell dimensions among cell populations were represented with box plots displaying the interquartile range (25^th^ and 75^th^ percentiles), the median value, and whiskers for minimum and maximum values. For nonparametric statistical analyses of our data, we performed U tests of Mann-Whitney^42^ using Prism7 (GraphPad), which provided the two-tailed distribution p-values indicated in Fig. 2 (with a critical value of 0.05).

### Electron microscopy analysis

Cells were grown at 37 °C in CY medium until OD_600_ = 0.3, and centrifuged at 3,220 × g for 10 min. A pellet volume of 1.4 μl was dispensed on the 200-μm side of a type A 3-mm gold platelet (Leica Microsystems), covered with the flat side of a type B 3-mm aluminum platelet (Leica Microsystems), and was vitrified by high-pressure freezing using an HPM100 system (Leica Microsystems). Next, the samples were freeze-substituted at −90 °C for 80 h in acetone supplemented with 1% OsO4 and warmed up slowly (1 °C/h) to −60 °C (AFS2; Leica Microsystems). After 8 to 12 h, the temperature was raised (1 °C/h) to −30 °C, and the samples were kept at this temperature for another 8 to 12 h before being rinsed 4 times in pure acetone. The samples were then infiltrated with gradually increasing concentrations of resin (Embed812, EMS) in acetone (1:2, 1:1, 2:1 [vol/vol]) for 3 h while raising the temperature to end at 20 °C. Pure resin was added at room temperature. After polymerization at 60 °C, 80-nm-thin sections were obtained using an ultramicrotome UC7 (Leica Microsystems) and were collected on formvar-carbon-coated 100-mesh copper grids. The thin sections were post stained for 5 min with 5% aqueous uranyl acetate, rinsed, and incubated for 2 min with lead citrate. The samples were observed using a CM12 (Philips) or Tecnai G2 Spirit BioTwin (FEI) microscope operating at 120 kV with an Orius SC1000B CCD camera (Gatan).

### Preparation of *S. pneumoniae* sacculi

Sacculi were prepared from wild-type (Spn5) and *Δpmp23* (spMJ26) *S. pneumoniae* cells as described previously^31^. Briefly, 2 l of culture in TH were harvested when OD_600_ reached 0.4–0.5 and resuspended in 40 ml of ice-cooled 50 mM Tris-HCl at pH 7.0. The cell suspension was poured dropwise into 150 ml of boiling 5% sodium dodecyl sulfate (SDS) solution and boiled for another 30 min. Sacculi were then harvested by centrifugation at room temperature and washed five times with water to remove SDS. Sacculi were disrupted with glass beads before treatment with DNaseI (10 µg/ml) and RNaseI (50 µg/ml) for 2 h at 37 °C in PBS supplemented with 20 mM MgSO_4_. This step was followed by addition of 10 mM CaCl_2_ and 100 µg/ml trypsin, and overnight incubation at 37 °C. The cell wall samples were boiled again in 1% SDS for 15 min, harvested by centrifugation at room temperature and washed several times with water before samples were stored in water at 4 °C.

For some of the *in vitro* Pmp23 activity assays, teichoic acid removal was performed by incubating the purified sacculi with 10% w/v aqueous trichloroacetic acid (TCA) overnight at 4 °C. The peptidoglycan fraction was pelleted by centrifugation (21,000 × g, 20 min, room temperature), washed and resuspended in water. Another batch of purified sacculi was chemically *N-*acetylated by addition of 0.25 volumes of 5% acetic anhydre and 0.25 volumes of saturated NaHCO_3_, incubation on ice for 30 min followed by 1 h at room temperature, as described by Vollmer and Tomasz^43^. The peptidoglycan was pelleted by centrifugation, washed and resuspended in water. To label sacculi with Remazol Brilliant Blue (RBB, Sigma-Aldrich), sacculi were incubated overnight at 37 °C with 20 mM RBB in 250 mM NaOH, followed by neutralization with HCl. The RBB-labeled sacculi were pelleted by centrifugation and washed with water until the supernatant was clear. The final pellet was resuspended in 1 ml of water and stored at 4 °C.

### Peptidoglycan digestion assays

Five µl of RBB-labeled sacculi were incubated overnight at 37 °C with 5 μM GST-Pmp23 or 4 μM mutanolysin, as a positive control, in 100 µl buffer A (50 mM Mes pH 6.0, 250 mM NaCl, 5 mM MgCl_2_, 0.5 mM DTT, 0.5 mM DDM). Reactions were terminated by incubation for 5 min at 95 °C. Soluble cleavage products were separated from intact peptidoglycan by centrifugation (21,000 × g for 10 min at room temperature). The supernatant was collected, and the absorbance was measured at 595 nm. Alternatively, 20 µl of unlabeled sacculi were incubated overnight at 37 °C with 5 μM GST-Pmp23 or 4 μM mutanolysin in 100 µl in buffer A. To monitor sacculi digestion, the absorbance was measured at 595 nm. Identical assays were performed on sacculi containing or not teichoic acids, and treated or not for chemical *N*-acetylation.

### Preparation of muropeptides and analysis of peptidoglycan composition

Experiments were performed as previously described^35^. In brief, cell wall samples were lyophilized overnight and incubated with 48% hydrofluoric acid for 48 h to remove secondary cell wall polymers. The resulting PG was digested with the muramidase cellosyl for 48 h at 37 °C with stirring. After cellosyl was inactivated by boiling for 10 min at 100 °C, the samples were reduced with sodium borohydride and the muropeptides were separated by HPLC (High-performance liquid chromatography) on a 250 × 4.6 mm 3 µm Prontosil 120-3-6 C18 AQ reversed-phase column (Bischoff, Leonberg, Germany). The eluted muropeptides were detected by their absorbance at 205 nm.

### Protein purification

To produce full-length Pmp23, two-liter cultures of *E. coli* BL21 (DE3) cells transformed with the pMJ18 plasmid (*pGex4T1:gst-pmp23*) were grown in Terrific Broth (BD Biosciences) at 37 °C until OD_600_ = 0.8. Protein production was induced with 0.5 mM IPTG (isopropyl β-D-thiogalactopyranoside) and cultures were further grown overnight at 16 °C. Cells were harvested by centrifugation and resuspended in 1/25^th^ volume buffer B (100 mM Mes pH 6.0, 500 mM NaCl, 10 mM MgCl_2_, 1 mM DTT,) containing the Complete^TM^ cocktail of protease inhibitor (Roche). Cell lysis was carried out using a Microfluidizer® M110-P (Microfluidics) at 10,000 psi and cell debris were pelleted by centrifugation at 40,000 × g for 30 min at 4 °C. The membrane fraction contained in the supernatant was separated by ultracentrifugation at 250,000 × g for 1 h at 4 °C. The membrane pellet was resuspended in 22 ml buffer B, solubilized with 10 mM of the nonionic detergent DDM (n-dodecyl-β-d-maltopyranoside, Sigma), and incubated overnight at 4 °C on a rotating wheel. After ultracentrifugation (250,000 × g, 1 h, 4 °C), the supernatant containing the solubilized membrane proteins was loaded on a Glutathione Sepharose affinity resin (GE Healthcare). After extensive wash, the protein was eluted in buffer C (50 mM Mes pH 6.0, 250 mM NaCl, 5 mM MgCl_2_, 0.5 mM DTT, 0.5 mM DDM, 10 mM reduced glutathione).

The GST-PBP2x recombinant construct was produced and purified as previously described^31^. Briefly, purification was performed in buffer D (50 mM Tris-HCl pH 8.0, 200 mM NaCl) using a Glutathione Sepharose affinity resin. After load and extensive wash, the resin was incubated with thrombin for 1 h at room temperature to cleave the GST and release PBP2x in the flow-through. Flow-through fractions were concentrated with Amicon Ultra centrifugation filter units (molecular weight cut-off of 30,000; Millipore) and injected onto a 10/300 GL Superdex 200 gel filtration column (GE Healthcare). Proteins were eluted in a buffer containing 25 mM Tris-HCl pH 8.0, 150 mM NaCl, and were concentrated again with Amicon Ultra centrifugation filter units.

The recombinant plasmid overproducing the extracellular domain His_7_-MapZ_extra2_^34^ was transformed into BL21(DE3) *E. coli* strain. The transformants were grown at 37 °C until the culture reached an OD_600_ = 0.6 in LB medium. Expression was induced by adding 0.5 mM IPTG and incubation was continued for 3 h. Proteins were purified on a Ni-NTA resin (Qiagen); the fractions corresponding to the pure protein were pooled and dialyzed overnight at 4 °C as described previously^34^. Protein concentration was determined using a Coomassie Assay Protein Dosage Reagent (Uptima) and aliquots were stored at −80 °C.

Protein concentrations were measured using absorbance at 280 nm.

### MapZ cell wall binding

Pneumococcal cell wall preparation as well as the procedure used to analyze MapZ binding to the cell wall was performed as previously described^31^. Briefly, 3 µg of purified His_7_-MapZ_extra2_ were incubated with or without purified *S. pneumoniae* cell wall (2.5 mg) in 300 μl of a buffer containing 50 mM Tris-HCl pH 8.0 and 100 mM NaCl for 16 h at 4 °C on a rotating platform. After centrifugation (5 min at 5,000 × g), the supernatant was removed (supernatant fraction S) and the cell wall pellet was washed three times with PBS and resuspended in 50 μl SDS-PAGE loading buffer. After incubation at 100 °C for 10 min, the supernatant, corresponding to His_7_-MapZ_extra2_ bound to the cell wall, was recovered from the cell wall pellet (pellet fraction P) by centrifugation (5 min at 5,000 × g). The different fractions were analysed by SDS-PAGE and Western immunoblotting. Detection of His_7_-MapZ_extra2_ was performed using mouse anti-histidine antibodies (Sigma). A goat anti-mouse secondary antibody HRP conjugate (Biorad) was used at 1/5,000 to reveal the immunoblots.

### MapZ phosphorylation analysis

After protein separation by 4–12% gradient SDS-PAGE, detection of MapZ phosphorylation in crude extracts of *S. pneumoniae* WT and *pmp23* mutant strains was performed by immunoblotting using an anti-phosphothreonine polyclonal antibody (Cell Signaling) at 1/2,000 as described previously^44^. A goat anti-rabbit secondary antibody HRP conjugate (Biorad) was used at 1/5,000 to reveal the immunoblots.

### Immunoprecipitation from detergent-solubilized membrane fractions

Immunoprecipitation experiments were adapted from^31^. One-liter cultures of *S. pneumoniae* strain R6 and spMJ15 were grown in TH medium containing 0.15 mM ZnCl_2_ for expression of *3xflag-pmp23*. Cells were harvested at OD_600_ = 0.3 by centrifugation (5,000 × g for 5 min at room temperature) and protoplasted by incubation at 37 °C for 3 h into 10 ml buffer A. The protoplasts were collected by centrifugation (8,000 × g for 10 min at 4 °C) and flash frozen in liquid N_2_. Thawed protoplasts were disrupted by osmotic lysis with 3 ml buffer B and membrane proteins were solubilized by addition of 2% Triton X-100. Incubation was carried out on a rotating wheel for 2 h at 4 °C. Cell debris were pelleted by ultracentrifugation (140,000 × g for 30 min at 4 °C) and the supernatant containing solubilized membrane proteins was collected. For each immunoprecipitation experiment, 500 μl of solubilized membrane proteins were incubated with 10 μl of anti-FLAG agarose resin (Sigma). After 5 h of incubation at 4 °C with gentle shaking, the resin was washed three times with buffer B-2% Triton before elution with 75 μl of FLAG peptide (Sigma). Proteins were separated by 12.5% SDS-PAGE and subjected to western blotting using appropriate anti-FLAG and anti-PBP2x sera dilutions.

### Microscale thermophoresis

Binding experiments were carried out with a Monolith NT.115 Series instrument (Nano Temper Technologies GMBH). GST-Pmp23 was labeled with the red dye NT-647. Four μl of sample containing 100 nM of labeled Pmp23 and increasing concentrations of PBP2x (from 7 nM to 235 μM) or BSA (negative control, from 5 nM to 360 μM) were loaded on K003 Monolith NT.115 hydrophilic treated silicon capillaries and thermophoresis was measured for 30 s. Each measurement was made in triplicates. Experiments were carried out at 25 °C in MST optimized buffer (50 mM Tris-HCl, 150 mM NaCl, 10 mM MgCl_2_, 0.05% Tween-20). Analysis was performed with the Monolith software. Affinity K_D_ was quantified by analyzing the change in normalized fluorescence (Fnorm = fluorescence after thermophoresis/initial fluorescence) as a function of the concentration of the PBP2x protein.

## Electronic supplementary material

Supplementary information

## References

[1] Vollmer W, Blanot D, de Pedro MA (2008). Peptidoglycan structure and architecture. FEMS Microbiol. Rev..

[2] Meeske AJ (2016). SEDS proteins are a widespread family of bacterial cell wall polymerases. Nature.

[3] Sauvage E, Kerff F, Terrak M, Ayala JA, Charlier P (2008). The penicillin-binding proteins: structure and role in peptidoglycan biosynthesis. FEMS Microbiol. Rev..

[4] Vollmer W, Joris B, Charlier P, Foster S (2008). Bacterial peptidoglycan (murein) hydrolases. FEMS Microbiol. Rev..

[5] Heijenoort J (2011). van. Peptidoglycan Hydrolases of *Escherichia coli*. Microbiol. Mol. Biol. Rev..

[6] Höltje J-V (1998). Growth of the Stress-Bearing and Shape-Maintaining Murein Sacculus of *Escherichia coli*. Microbiol. Mol. Biol. Rev..

[7] Haeusser DP, Margolin W (2016). Splitsville: structural and functional insights into the dynamic bacterial Z ring. Nat. Rev. Microbiol..

[8] Sham L-T, Tsui H-CT, Land AD, Barendt SM, Winkler ME (2012). Recent advances in pneumococcal peptidoglycan biosynthesis suggest new vaccine and antimicrobial targets. Curr. Opin. Microbiol..

[9] Egan AJF, Vollmer W (2013). The physiology of bacterial cell division. Ann. N. Y. Acad. Sci..

[10] Massidda O, Nováková L, Vollmer W (2013). From models to pathogens: how much have we learned about *Streptococcus pneumoniae* cell division?. Environ. Microbiol..

[11] Fleurie A (2014). Interplay of the Serine/Threonine-Kinase StkP and the Paralogs DivIVA and GpsB in Pneumococcal Cell Elongation and Division. PLOS Genet.

[12] Fleurie A (2014). MapZ marks the division sites and positions FtsZ rings in *Streptococcus pneumoniae*. Nature.

[13] Holečková N (2015). LocZ Is a New Cell Division Protein Involved in Proper Septum Placement in *Streptococcus pneumoniae*. mBio.

[14] van Raaphorst R, Kjos M, Veening J-W (2017). Chromosome segregation drives division site selection in *Streptococcus pneumoniae*. Proc. Natl. Acad. Sci. USA.

[15] Schuster C, Dobrinski B, Hakenbeck R (1990). Unusual septum formation in *Streptococcus pneumoniae* mutants with an alteration in the D,D-carboxypeptidase penicillin-binding protein 3. J. Bacteriol..

[16] Morlot C, Noirclerc-Savoye M, Zapun A, Dideberg O, Vernet T (2004). The d,d-carboxypeptidase PBP3 organizes the division process of *Streptococcus pneumoniae*. Mol. Microbiol..

[17] Ng W-L, Kazmierczak KM, Winkler ME (2004). Defective cell wall synthesis in *Streptococcus pneumoniae* R6 depleted for the essential PcsB putative murein hydrolase or the VicR (YycF) response regulator. Mol. Microbiol..

[18] Pagliero E (2008). The inactivation of a new peptidoglycan hydrolase Pmp23 leads to abnormal septum formation in *Streptococcus pneumoniae*. Open Microbiol. J..

[19] Barendt SM, Sham L-T, Winkler ME (2011). Characterization of Mutants Deficient in the l,d-Carboxypeptidase (DacB) and WalRK (VicRK) Regulon, Involved in Peptidoglycan Maturation of *Streptococcus pneumoniae* Serotype 2 Strain D39. J. Bacteriol..

[20] Tsui H-CT (2016). Suppression of a deletion mutation in the gene encoding essential PBP2b reveals a new lytic transglycosylase involved in peripheral peptidoglycan synthesis in *Streptococcus pneumoniae* D39. Mol. Microbiol..

[21] Bartual SG (2014). Structural basis of PcsB-mediated cell separation in *Streptococcus pneumoniae*. Nat. Commun..

[22] Morlot C (2005). Crystal Structure of a Peptidoglycan Synthesis Regulatory Factor (PBP3) from *Streptococcus pneumoniae*. J. Biol. Chem..

[23] Abdullah MR (2014). Structure of the pneumococcal l,d-carboxypeptidase DacB and pathophysiological effects of disabled cell wall hydrolases DacA and DacB. Mol. Microbiol..

[24] Hoyland CN (2014). Structure of the LdcB LD-Carboxypeptidase Reveals the Molecular Basis of Peptidoglycan Recognition. Structure.

[25] Rivas BDL, García JL, López R, García P (2002). Purification and Polar Localization of Pneumococcal LytB, a Putative Endo-β-N-Acetylglucosaminidase: the Chain-Dispersing Murein Hydrolase. J. Bacteriol..

[26] Pagliero E, Dideberg O, Vernet T, Guilmi AMD (2005). The PECACE domain: a new family of enzymes with potential peptidoglycan cleavage activity in Gram-positive bacteria. BMC Genomics.

[27] Martín-Galiano AJ, Yuste J, Cercenado MI, de la Campa AG (2014). Inspecting the potential physiological and biomedical value of 44 conserved uncharacterised proteins of *Streptococcus pneumoniae*. BMC Genomics.

[28] Fukushima T (2008). Identification and Characterization of Novel Cell Wall Hydrolase CwlT: a two-domain autolysin exhibiting N-acetylmuramidase and DL-endopeptidase activities. J. Biol. Chem..

[29] Xu Q (2014). Structures of a Bifunctional Cell Wall Hydrolase CwlT Containing a Novel Bacterial Lysozyme and an NlpC/P60 dl-Endopeptidase. J. Mol. Biol..

[30] Morlot C, Zapun A, Dideberg O, Vernet T (2003). Growth and division of *Streptococcus pneumoniae*: localization of the high molecular weight penicillin-binding proteins during the cell cycle. Mol. Microbiol..

[31] Morlot C (2013). Interaction of Penicillin-Binding Protein 2x and Ser/Thr protein kinase StkP, two key players in *Streptococcus pneumoniae* R6 morphogenesis. Mol. Microbiol..

[32] Zapun A, Vernet T, Pinho MG (2008). The different shapes of cocci. FEMS Microbiol. Rev..

[33] Kuru E (2012). In Situ Probing of Newly Synthesized Peptidoglycan in Live Bacteria with Fluorescent D-Amino Acids. Angew. Chem. Int. Ed..

[34] Manuse S (2016). Structure–function analysis of the extracellular domain of the pneumococcal cell division site positioning protein MapZ. Nat. Commun..

[35] Bui NK (2012). Isolation and analysis of cell wall components from *Streptococcus pneumoniae*. Anal. Biochem..

[36] Wheeler R, Mesnage S, Boneca IG, Hobbs JK, Foster SJ (2011). Super-resolution microscopy reveals cell wall dynamics and peptidoglycan architecture in ovococcal bacteria. Mol. Microbiol..

[37] Tsui H-CT (2014). Pbp2x localizes separately from Pbp2b and other peptidoglycan synthesis proteins during later stages of cell division of *Streptococcus pneumoniae* D39. Mol. Microbiol..

[38] Potluri L-P, de Pedro MA, Young KD (2012). *Escherichia coli* low-molecular-weight penicillin-binding proteins help orient septal FtsZ, and their absence leads to asymmetric cell division and branching. Mol. Microbiol..

[39] Lacks S, Hotchkiss RD (1960). A study of the genetic material determining an enzyme in Pneumococcus. Biochim. Biophys. Acta.

[40] Sung CK, Li H, Claverys JP, Morrison DA (2001). An rpsL Cassette, Janus, for Gene Replacement through Negative Selection in *Streptococcus pneumoniae*. Appl. Environ. Microbiol..

[41] Sliusarenko O, Heinritz J, Emonet T, Jacobs-Wagner C (2011). High-throughput, subpixel precision analysis of bacterial morphogenesis and intracellular spatio-temporal dynamics. Mol. Microbiol..

[42] Mann HB, Whitney D (1947). R. On a Test of Whether one of Two Random Variables is Stochastically Larger than the Other. Ann. Math. Stat..

[43] Vollmer W, Tomasz A (2000). The pgdA Gene Encodes for a PeptidoglycanN-Acetylglucosamine Deacetylase in *Streptococcus pneumoniae*. J. Biol. Chem..

[44] Fleurie A (2012). Mutational dissection of the S/T-kinase StkP reveals crucial roles in cell division of *Streptococcus pneumoniae*. Mol. Microbiol..

