# Supplementary material for: The cell wall hydrolase Pmp23 is important for assembly and stability of the division ring in Streptococcus pneumoniae
Source: Sci Rep. 2018 May 15;8:7591. doi: 10.1038/s41598-018-25882-y (PMC5954120; doi:10.1038/s41598-018-25882-y)
Supplement: Supplementary file 1 — Supplementary information [file 41598_2018_25882_MOESM1_ESM.pdf]

The cell wall hydrolase Pmp23 is important for assembly and stability of the division ring in *Streptococcus pneumoniae*

Maxime Jacq, Christopher Arthaud, Sylvie Manuse, Chryslène Mercy, Laure Bellard, Katharina Peters, Benoit Gallet, Jennifer Galindo, Thierry Doan, Waldemar Vollmer, Yves V. Brun, Michael S. VanNieuwenhze, Anne Marie Di Guilmi, Thierry Vernet, Christophe Grangeasse and Cecile Morlot

Supplementary Information

Supplementary figure legends

Figure S1. Sequence analysis of Pmp23 and comparison of its homology model with bacterial lysozyme structures. a. The sequence of Pmp23 from *S. pneumoniae* R6 was aligned with MltE from *E. coli* or with the G-type lysozyme from *Gadus morhua* (G-type lyso). Conserved residues are in red boxes, similar residues in yellow boxed red characters. Residue numbering is for Pmp23. The catalytic Glu and Asp residues of bLysG domains are highlighted with red stars. Note that both residues are present in Pmp23 (E61 and D68), but that the Asp is not conserved in MltE and the G-type lysozyme. The lysozyme-specific Asn and Glu/Asp residues, which are only present in Pmp23 (N119 and E74) and the G-type lysozyme, are highlighted with blue stars. The bLysG-specific DVMQSSES motif is highlighted with an orange box. **b-c.** Alternative views of the surface representation of the Pmp23 homology models ($Pmp23_{4HPE}$ and $Pmp23_{4FDY}$) and comparison with their CwlT templates from *C. difficile* ($CwlT_{Cd}$, PDB code 4HPE) and *S. aureus* ($CwlT_{Sa}$, PDB code 4FDY). The Glu and Asp residues required for CwlT activity are labeled and colored red. The DVMQSSES motif is colored in orange. The positions of the C-terminal and N-terminal lobes are indicated.

Figure S2. Characterization of *S. pneumoniae* morphology when Pmp23 is inactivated. a. Immunoblot analysis of whole-cell lysates from a *S. pneumoniae* R6 strain and strains expressing wild-type (WT), E61Q and D68N variants of *sfgfp-pmp23* with anti-GFP antibodies (upper panel). Enolase levels were monitored to control for loading (lower panel). **b-e.** Electron microscopy images of thin sections of wild-type (**b**), *Δpmp23* (**c**), *pmp23*(E61Q) (**d**) and *pmp23*(D68N) (**e**) *S. pneumoniae* cells. Scale bars = 200 nm.

Figure S3. FtsZ localization in the presence of Pmp23 or when Pmp23 is inactive. a. Live *S. pneumoniae* cells expressing an endogenous *ftsZ-gfp* fusion in wild-type (*WT*), *Δpmp23* and *pmp23*(E61Q) genetic backgrounds. Examples of cells with wild-type FtsZ localization (green arrows) and distorted cells with asymmetric (orange arrowheads) or helical (orange arrows) FtsZ localization are indicated. Phase contrast (PC), GFP fluorescence and merged images are shown. Scale bars = 1 µm. **b.** Immunoblot analysis of whole-cell lysates from wild-type (*WT*), *Δpmp23* and *pmp23*(E61Q) *S. pneumoniae* strains expressing an endogenous *ftsZ-gfp* fusion with anti-FtsZ antibodies. Enolase levels were monitored to control for loading. **c.** Native FtsZ was labeled in fixed wild-type (*WT*), *Δpmp23* and *pmp23*(E61Q) cells using mouse anti-FtsZ serum and Cy2-conjugated anti-mouse IgG. Examples of cells with wild-type FtsZ localization (green arrows) and distorted cells with helical (orange arrows) FtsZ localization are indicated. GFP fluorescence and merged images (between the Cy2 and the phase contrast channels) are shown. Scale bars = 1 µm.

Figure S4. Time-lapse FtsZ localization in *pmp23*(E61Q) mutant cells. Live *S. pneumoniae* cells expressing an endogenous *ftsZ-gfp* fusion in the *pmp23*(E61Q) genetic background were grown in CH medium at 30°C. Orange arrowheads point to asymmetric Z-ring positioning and white arrowheads indicate cells undergoing active asymmetric division. Orange arrows indicate helical FtsZ localizations appearing as soon as FtsZ is detected in the newborn cell and blue arrows indicate helical FtsZ localization arising from a single Z-ring. White arrows point at cells undergoing active division despite helical FtsZ localization. Phase contrast (PC), GFP fluorescence and merged images are shown. Scale bars = 1 µm.

Figure S5. MapZ localization in the presence of Pmp23 or when Pmp23 is inactive. a. Live *S. pneumoniae* cells expressing an endogenous *gfp-mapZ* fusion in wild-type (WT), *Δpmp23* and *pmp23*(E61Q) genetic backgrounds. Arrows point to cells displaying wild-type morphology and wild-type MapZ localizations (green), enlarged cells with a slight membrane delocalization of MapZ (yellow) and abnormal morphology with aberrant MapZ localizations (orange). Phase contrast (PC), GFP fluorescence and merged images are shown. Scale bars = 1 μm. **b.** Immunoblot analysis of whole-cell lysates from wild-type (WT), *Δpmp23* and *pmp23*(E61Q) *S. pneumoniae* strains expressing an endogenous *gfp-mapZ* fusion with anti-MapZ antibodies. Enolase levels were monitored to control for loading.

Figure S6. Peptidoglycan modifications in the absence of Pmp23. a. Representative muropeptide profiles from *S. pneumoniae* wild-type (*WT*) and *Δpmp23* cells obtained by reversed-phase HPLC. Peaks were assigned to muropeptides according to their retention times as previously published[35]. **b.** Table showing the proportion of monomers, dimers, trimers and the percent of peptides in crosslinks (calculated as 100% - % monomers) in purified PG from wild-type and *Δpmp23* cells. The values are mean ± variance of two biological replicates.

Figure S7. MapZ binding to peptidoglycan, phosphorylation and StkP septal localization are not affected by the absence of Pmp23. a. His_7-$MapZ_{extra2}$ was incubated at 4°C for 16 h in the presence (left panel) or in the absence (right panel, NP for no peptidoglycan) of purified sacculi from wild-type (WT), *Δpmp23* or *pmp23*(E61Q) cells. The protein fraction bound to the cell wall was separated from the unbound fraction by centrifugation. For each experiment, the supernatant (S) and pellet (P) fractions from the centrifugation were analyzed by SDS-PAGE on a 12.5%

polyacrylamide gel and immunoblotted using anti-histidine antibodies. **b.** After protein separation by SDS-PAGE on a 4-12% gradient polyacrylamide gel, immunoblot analysis of whole-cell lysates from wild-type (WT), *Δpmp23* or *pmp23*(E61Q) *S. pneumoniae* R6 strains using anti-phosphothreonine antibodies shows equivalent phosphorylation signals for MapZ. **c.** Live *S. pneumoniae* cells expressing an endogenous *gfp-stkp* fusion in wild-type (*WT*) and *Δpmp23* genetic backgrounds. Examples of septal StkP localization patterns in *Δpmp23* cells displaying wild-type morphology (green arrows) or abnormal morphology (orange arrows) are indicated. Phase contrast (PC), GFP fluorescence and merged images are shown. Scale bars = 1 μm.

Figure S8. Full-size images of the immunoblots displayed throughout the manuscript. Legends are identical to those of panels 6b **(a)**, S2a **(b)**, S3b **(c)**, S5b **(d)**, S7a **(e)** and S7b **(f)**.

Table S1. Strains and plasmids used in this study.

Table S2. Oligonucleotide primers used in this study.

Figure S1

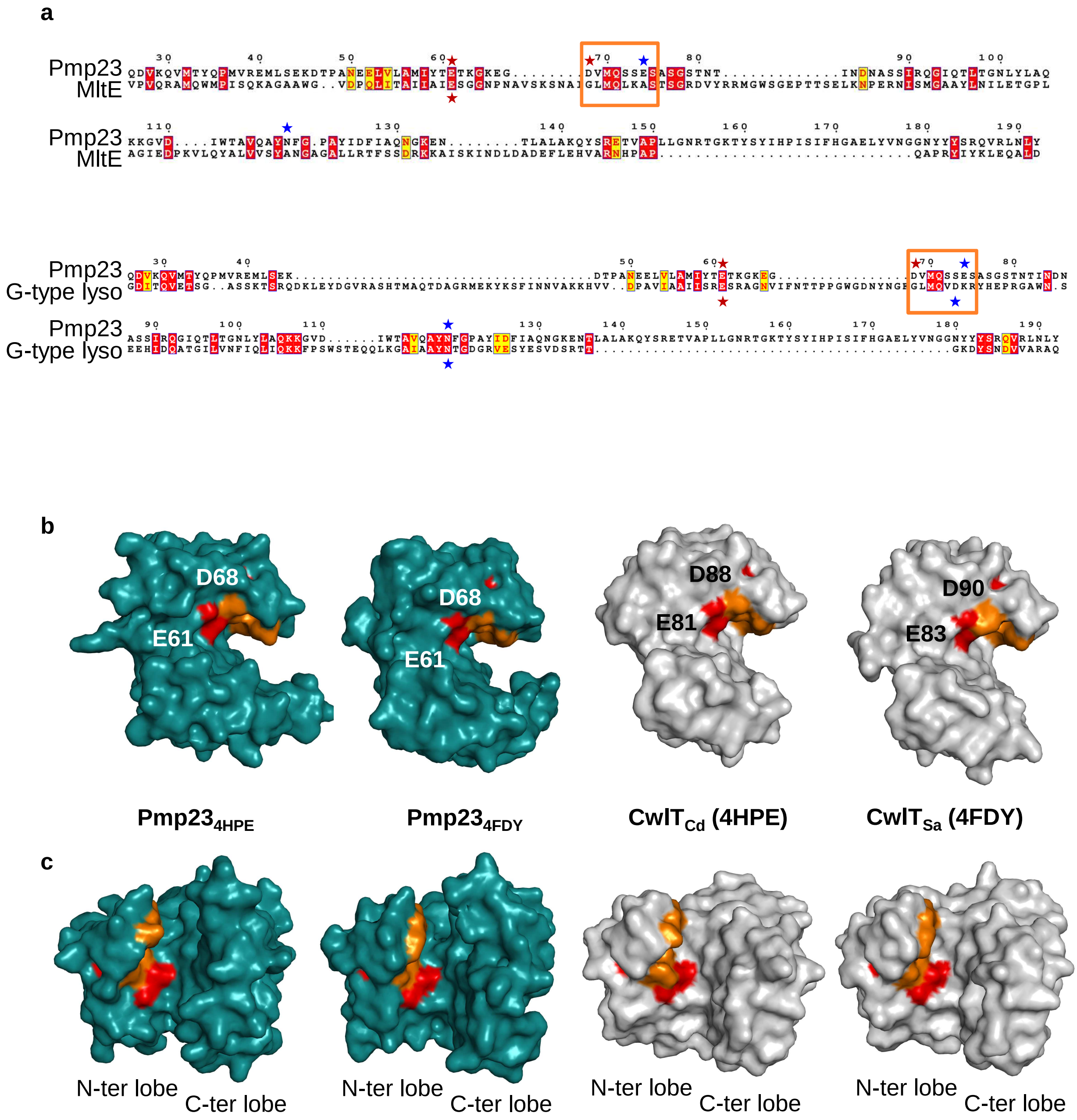
a
Pmp23
MltE
Pmp23
MltE
Pmp23
G-type lyso
Pmp23
G-type lyso
b
D68
E61
D68
E61
D88
E81
D90
E83
Pmp23$_{4HPE}$
Pmp23$_{4FDY}$
CwlT$_{Cd}$ (4HPE)
CwlT$_{Sa}$ (4FDY)
c
N-ter lobe
C-ter lobe
N-ter lobe
C-ter lobe
N-ter lobe
C-ter lobe
N-ter lobe
C-ter lobe

Figure S2

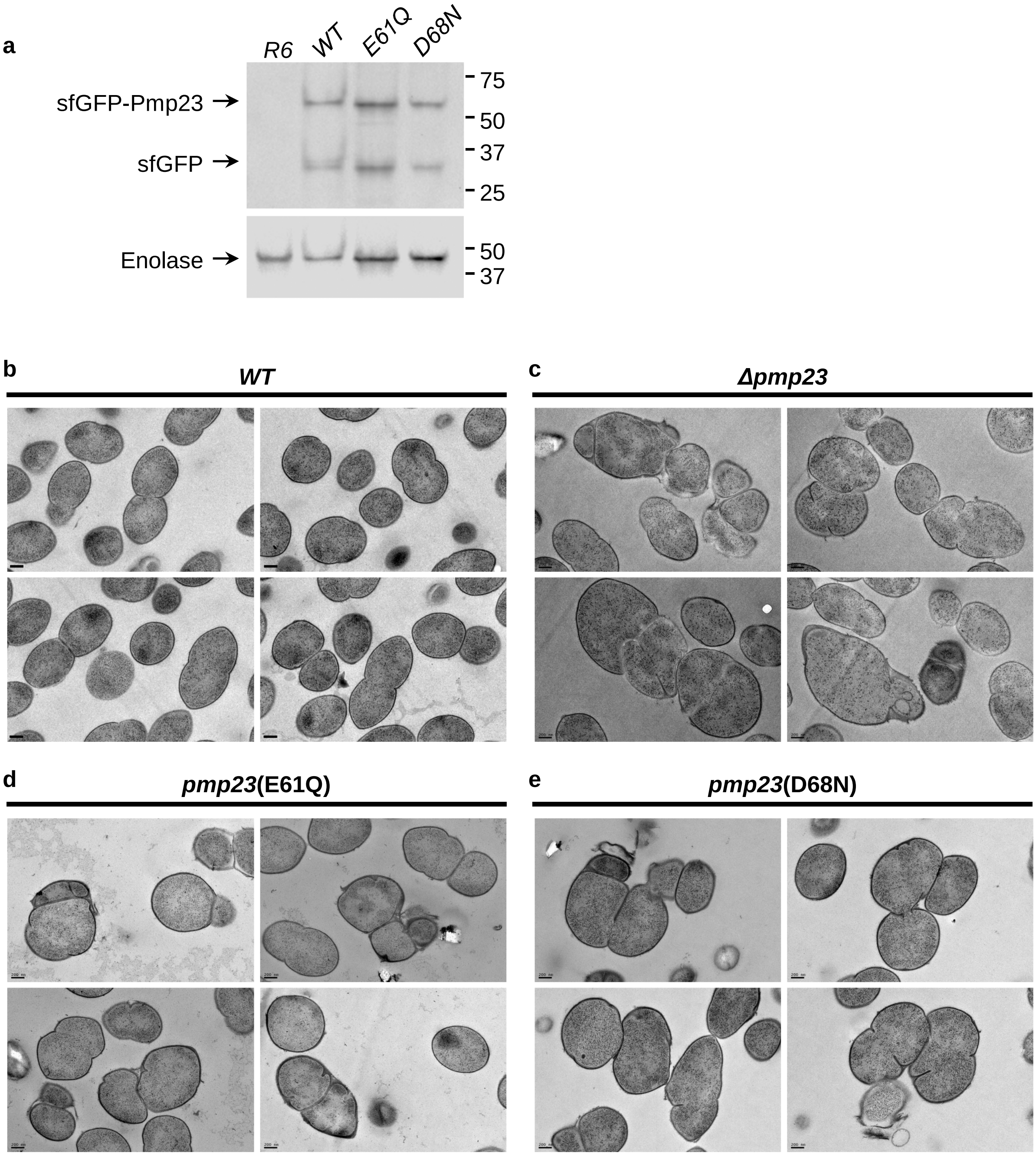
a
R6
WT
E61Q
D68N
sfGFP-Pmp23
sfGFP
Enolase
75
50
37
25
50
37
b
WT
c
Δpmp23
d
pmp23(E61Q)
e
pmp23(D68N)

Figure S3

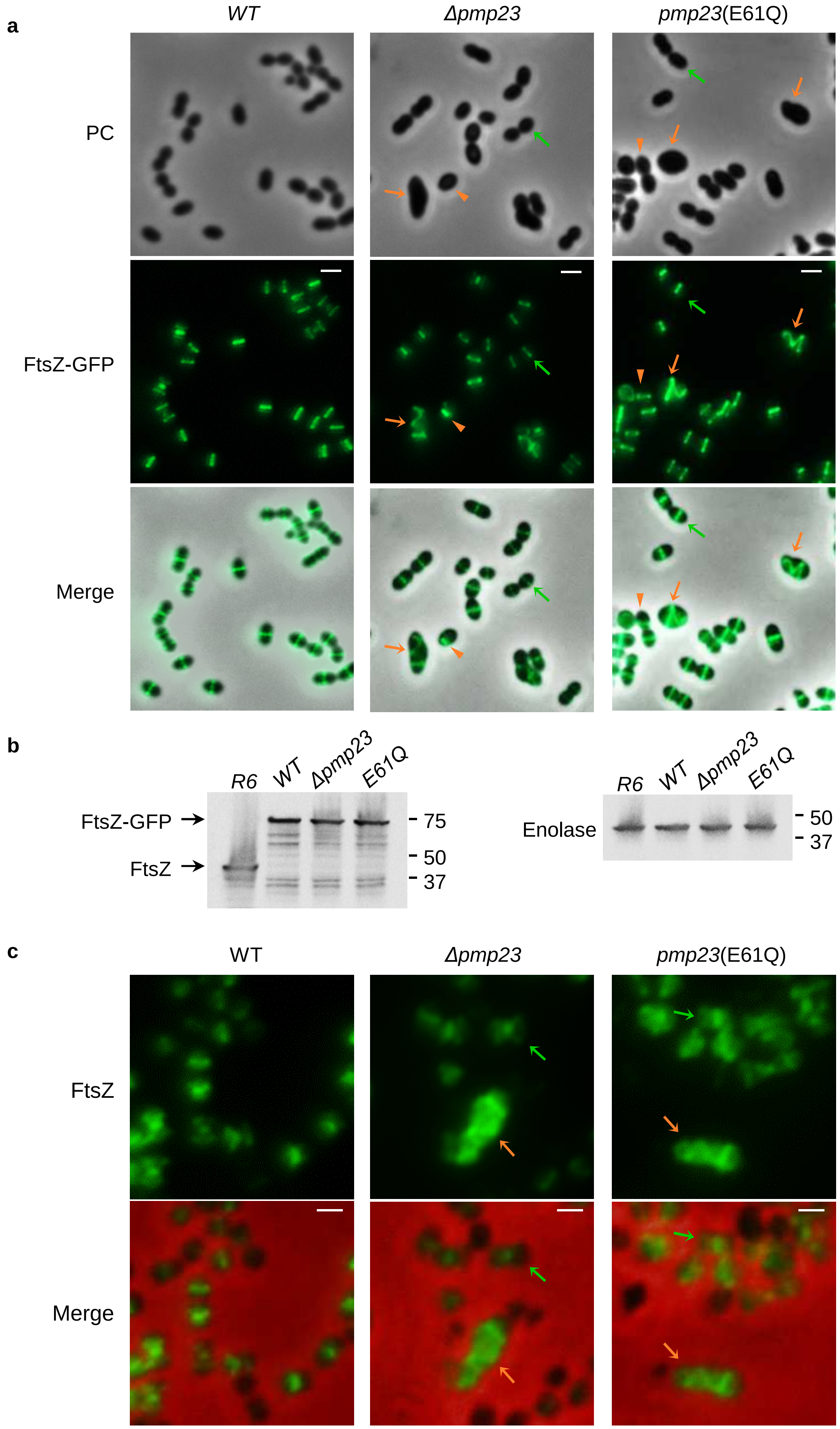
a
WT
Δpmp23
pmp23(E61Q)
PC
FtsZ-GFP
Merge
b
R6
WT
Δpmp23
E61Q
FtsZ-GFP
FtsZ
75
50
37
Enolase
50
37
c
WT
Δpmp23
pmp23(E61Q)
FtsZ
Merge

Figure S4

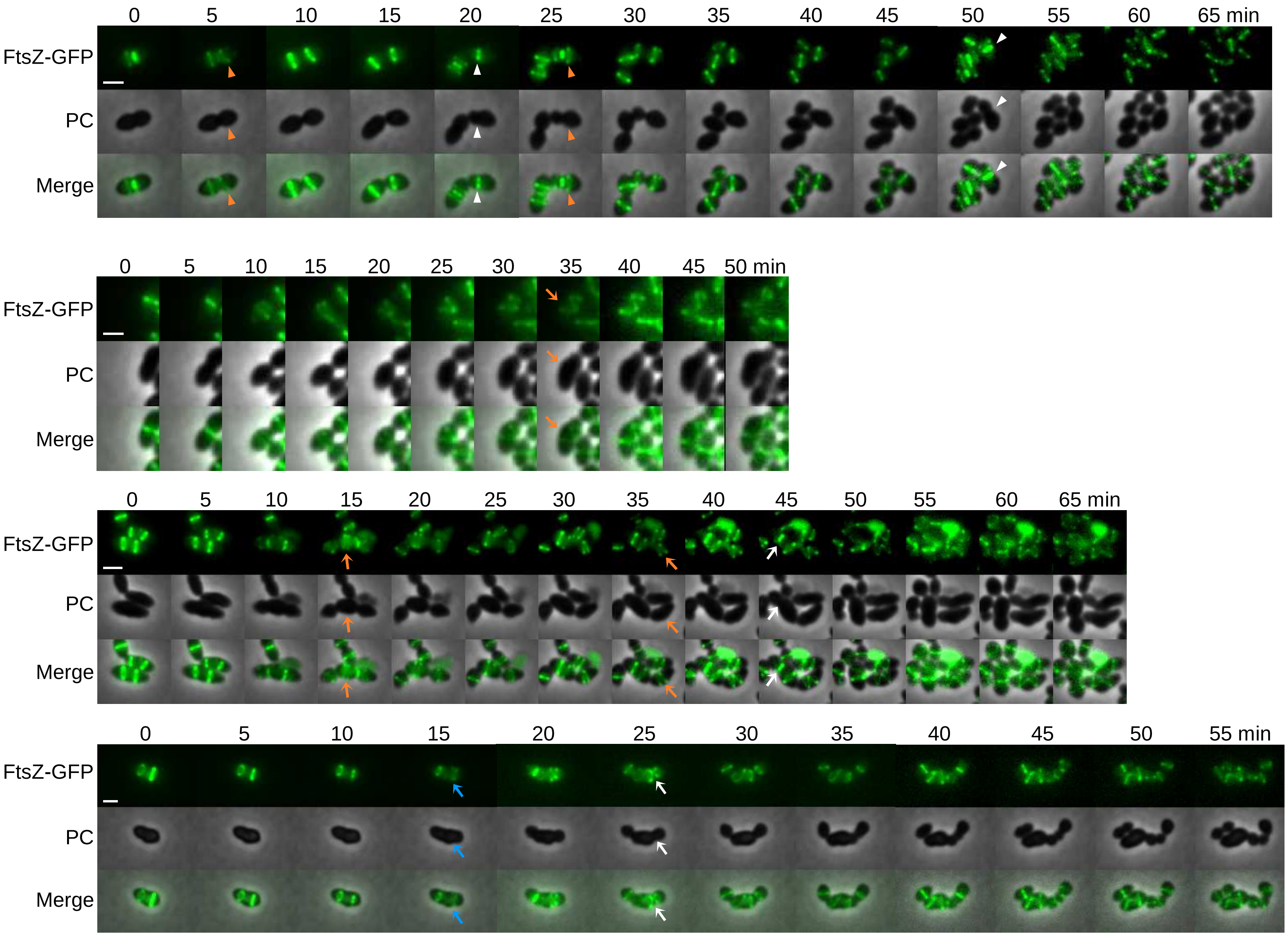
0
5
10
15
20
25
30
35
40
45
50
55
60
65 min
FtsZ-GFP
PC
Merge
0
5
10
15
20
25
30
35
40
45
50 min
FtsZ-GFP
PC
Merge
0
5
10
15
20
25
30
35
40
45
50
55
60
65 min
FtsZ-GFP
PC
Merge
0
5
10
15
20
25
30
35
40
45
50
55 min
FtsZ-GFP
PC
Merge

Figure S5

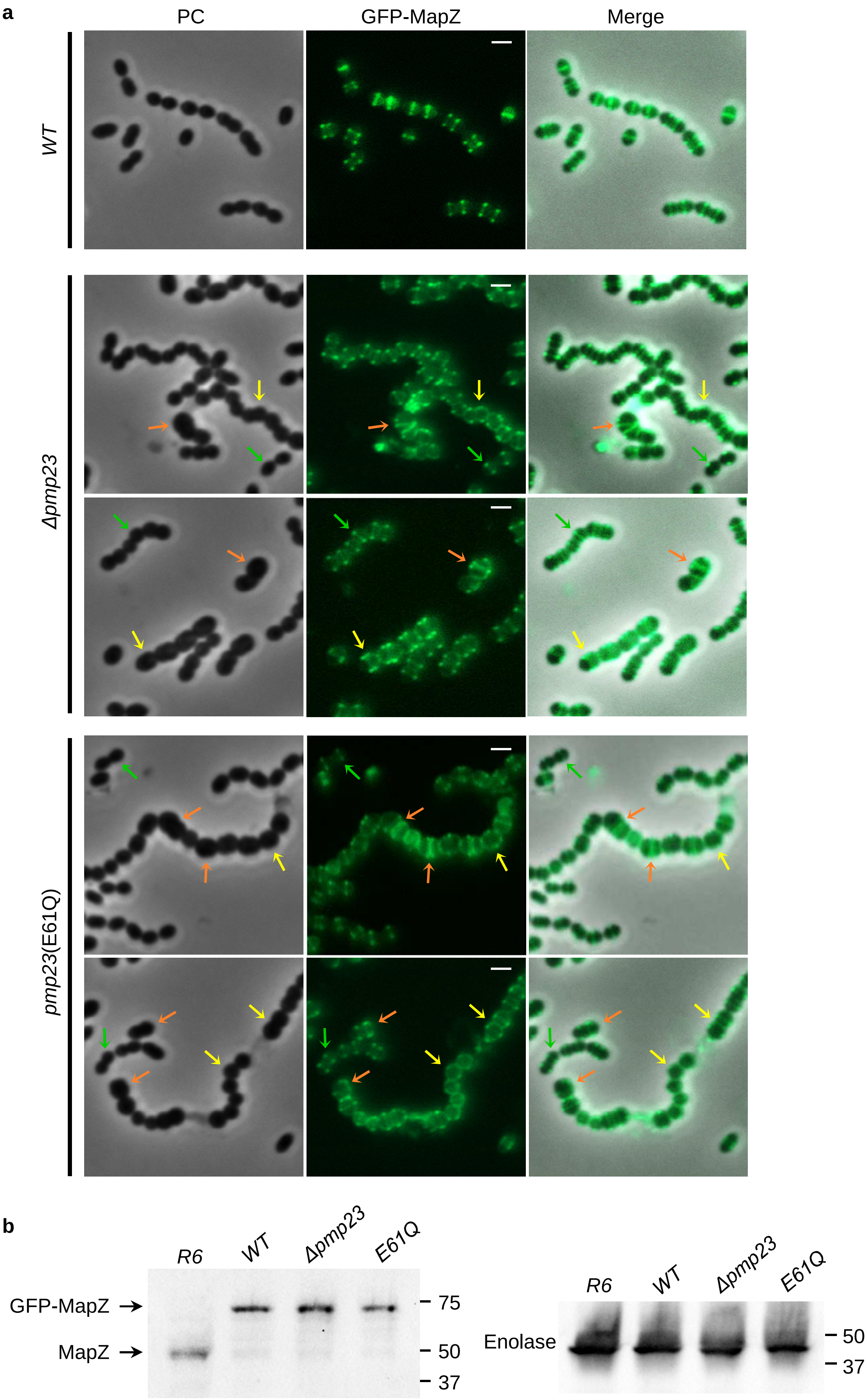
a
PC
GFP-MapZ
Merge
WT
Δpmp23
pmp23(E61Q)
b
R6
WT
Δpmp23
E61Q
GFP-MapZ
MapZ
75
50
37
Enolase
50
37

Figure S6

a

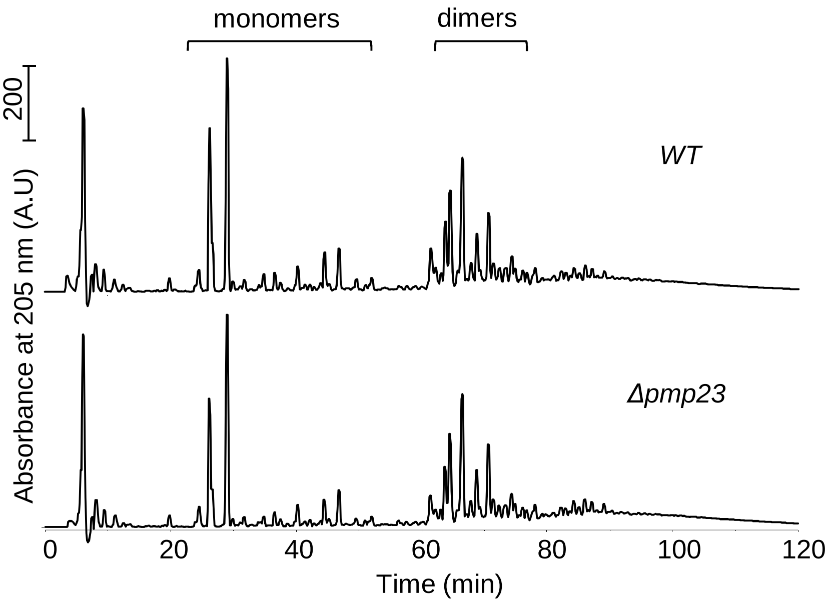

b

PG feature	Relative %	
	WT	*Δpmp23*
Monomers	49.8 ± 0.7	45.7 ± 2.0
Dimers	49.0 ± 0.7	53.1 ± 2.1
Trimers	1.2 ± 0.1	1.2 ± 0.2
Peptides in crosslinks	50.3 ± 0.7	54.3 ± 2.0

Figure S7

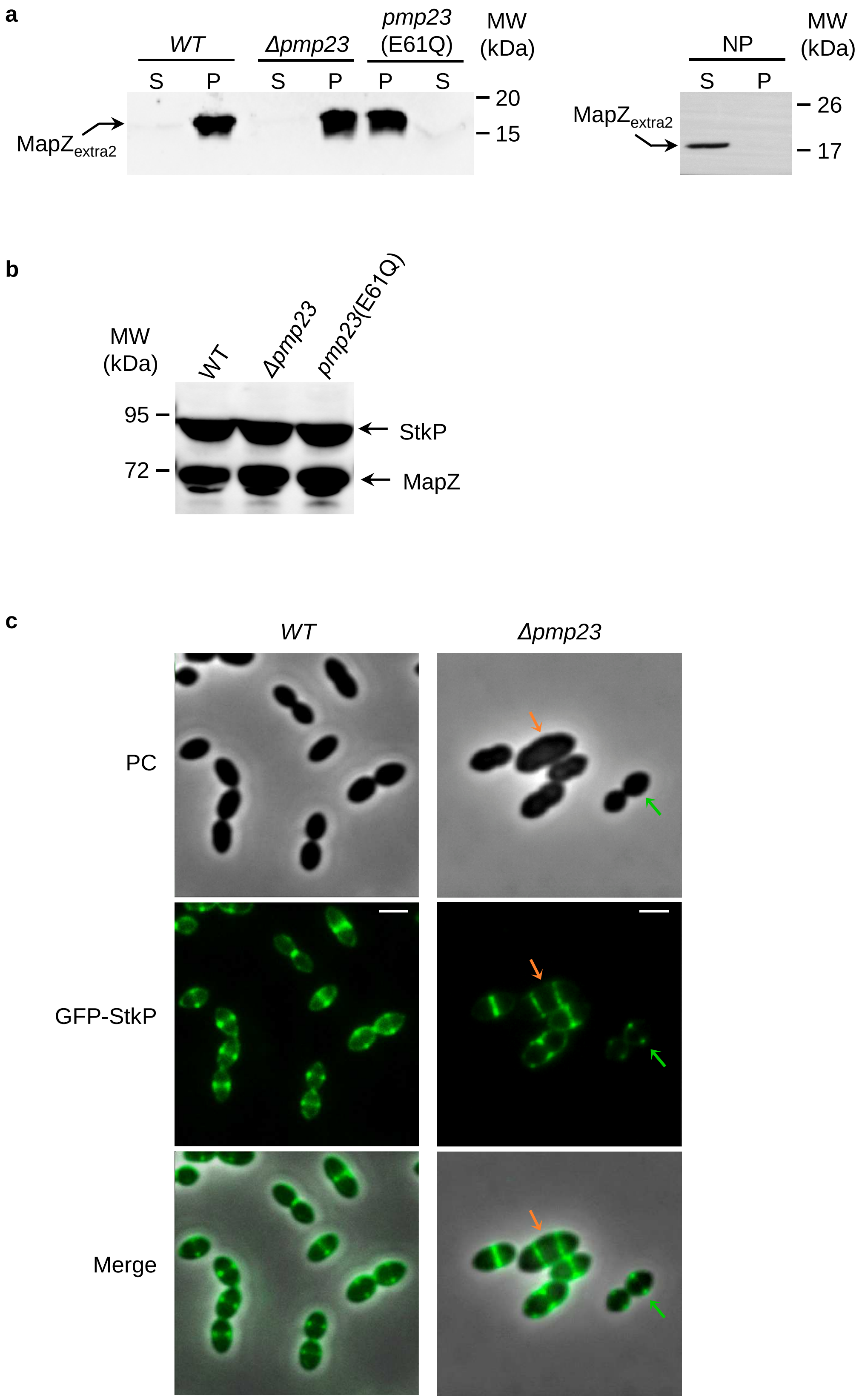
a
WT
Δpmp23
pmp23 (E61Q)
MW (kDa)
S P S P P S
20
15
MapZextra2
NP
MW (kDa)
S P
26
17
MapZextra2
b
MW (kDa)
WT
Δpmp23
pmp23(E61Q)
95
72
StkP
MapZ
c
WT
Δpmp23
PC
GFP-StkP
Merge

Figure S8

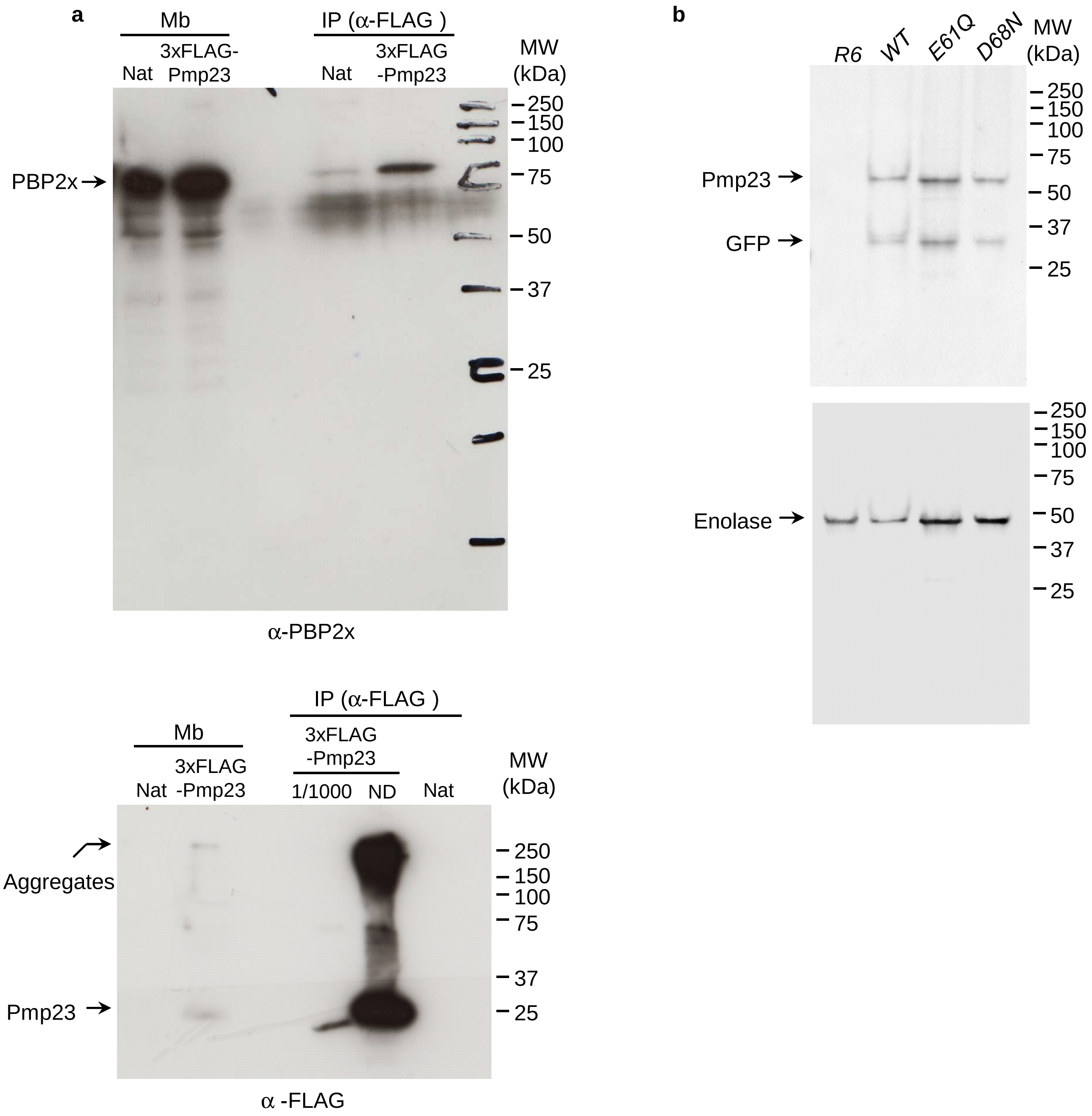
a
Mb
Nat
3xFLAG-Pmp23
IP (α-FLAG)
Nat
3xFLAG -Pmp23
MW (kDa)
250
150
100
75
50
37
25
PBP2x
α-PBP2x
Mb
Nat
3xFLAG -Pmp23
IP (α-FLAG)
3xFLAG -Pmp23
1/1000
ND
Nat
MW (kDa)
Aggregates
Pmp23
250
150
100
75
37
25
α -FLAG
b
R6
WT
E61Q
D68N
MW (kDa)
Pmp23
GFP
250
150
100
75
50
37
25
Enolase
250
150
100
75
50
37
25

Figure S8

c

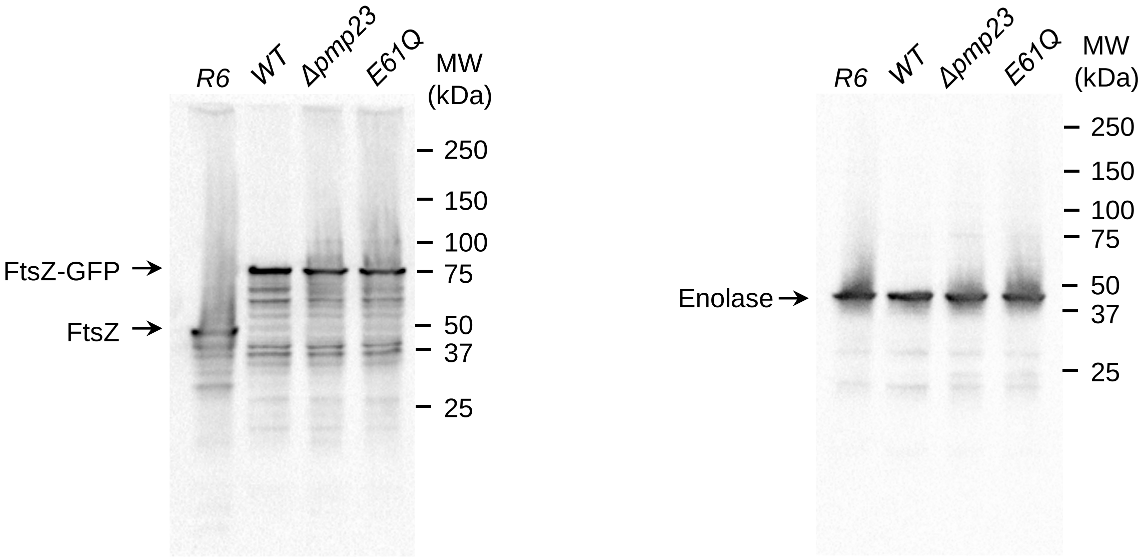

R6
WT
Δpmp23
E61Q
MW (kDa)
250
150
100
75
50
37
25
FtsZ-GFP
FtsZ
Enolase

d

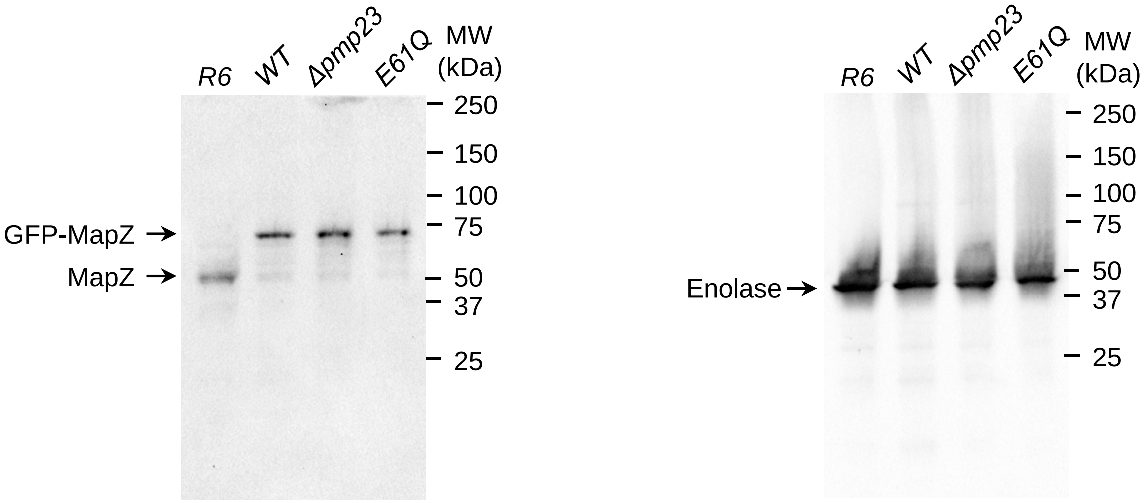

R6
WT
Δpmp23
E61Q
MW (kDa)
250
150
100
75
50
37
25
GFP-MapZ
MapZ
Enolase

Figure S8

e

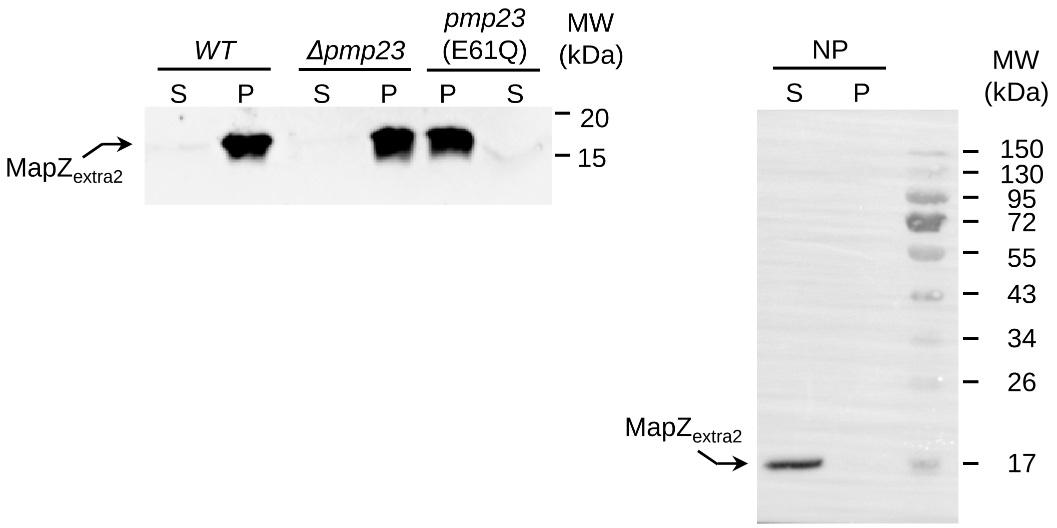
WT
Δpmp23
pmp23
(E61Q)
MW
(kDa)
S P S P P S
20
15
MapZextra2
NP
S P
MW
(kDa)
150
130
95
72
55
43
34
26
MapZextra2
17

f

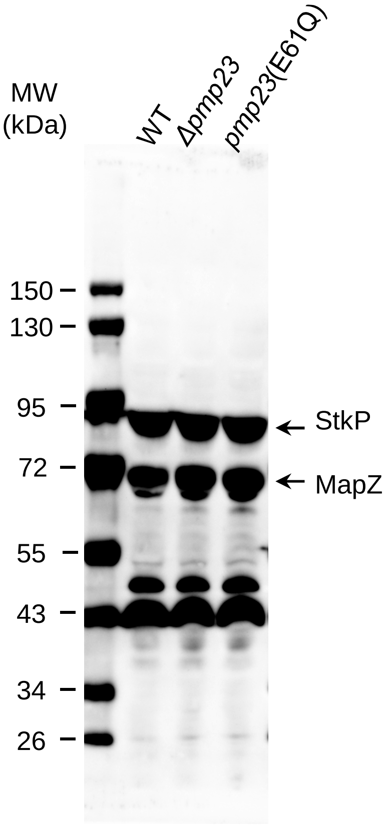
MW
(kDa)
WT
Δpmp23
pmp23(E61Q)
150
130
95
72
55
43
34
26
StkP
MapZ

Table S1. Strains and plasmids used in this study

Strains	Relevant genotype or description	Reference
E. coli		
DH5α	*F^- endA1 glnV44 thi-1 recA1 relA1 gyrA96 deoR nupG Φ80dlacZΔM15 Δ(lacZYA-argF)U169, hsdR17(r_K^- m_K^+), λ–*	1
BL21(DE3)	*F- ompT gal dcm lon hsdS$_B$ (r_B- m_B-) λ(*DE3 *[lac*I *lac*UV5-T7 *gene 1 ind*1 *sam*7 *nin*5])	2
S. pneumoniae		
R6	*S. pneumoniae* R36A derivative	3
Spn5 (WT)	R800 *rpsL1; Str^R*	4
spMJ96	Spn5 *pmp23::kan-rpsL; Kan^R*	This work
spMJ26	Spn5 *pmp23::Δpmp23; Str^R*	This work
spMJ98	Spn5 *pmp23::pmp23(E61Q); Str^R*	This work
spMJ86	Spn5 *pmp23::pmp23(D68N); Str^R*	This work
spMJ11	Spn5 *pmp23::Δpmp23; Str^R; bgaA::P$_{Zn}$-sfgfp-pmp23; Tet^R*	This work
spMJ7	R6 *bgaA::P$_{Zn}$-sfgfp-pmp23; Tet^R*	This work
spnLB32	R6 *bgaA::P$_{Zn}$-sfgfp-pmp23(E61Q); Tet^R*	This work
spnLB33	R6 *bgaA::P$_{Zn}$-sfgfp-pmp23(D68N); Tet^R*	This work
spMJ14	Spn5 *pmp23::Δpmp23; Str^R; bgaA::P$_{Zn}$-3xflag-pmp23; Tet^R*	This work
spMJ15	R6 *bgaA::P$_{Zn}$-3xflag-pmp23; Tet^R*	This work
spMJ39	Spn5 *ftsZ::ftsZ-gfp+; Str^R*	5
spMJ95	Spn5 *ftsZ::ftsZ-gfp+; pmp23::Δpmp23; Str^R*	This work
spMJ99	Spn5 *ftsZ::ftsZ-gfp+; pmp23::pmp23(E61Q); Str^R*	This work
spn195	Spn5 *mapZ::gfp+-mapZ; Str^R*	6
spMJ106	Spn5 *mapZ::gfp+-mapZ; pmp23::Δpmp23; Str^R*	This work
spMJ107	Spn5 *mapZ::gfp+-mapZ; pmp23::pmp23(E61Q); Str^R*	This work
spn476	Spn5 *ftsZ::ftsZ-mKate2, pbp2x::gfp-pbp2x; Str^R*	5
spMJ101	Spn5 *ftsZ::ftsZ-mKate2, pbp2x::gfp-pbp2x; pmp23::Δpmp23; Str^R*	This work
spMJ104	Spn5 *ftsZ::ftsZ-mKate2, pbp2x::gfp-pbp2x; pmp23::pmp23(E61Q); Str^R*	This work
spn479	Spn5 *ftsZ::ftsZ-mKate2, pbp2b::gfp-pbp2b; Str^R*	5
spMJ103	Spn5 *ftsZ::ftsZ-mKate2, pbp2b::gfp-pbp2b; pmp23::Δpmp23; Str^R*	This work
spMJ105	Spn5 *ftsZ::ftsZ-mKate2, pbp2b::gfp-pbp2b; pmp23::pmp23(E61Q); Str^R*	This work
Spn110	Spn5 *stkP::gfp-stkP; Str^R*	7
Spn1377	Spn5 *stkP::gfp-stkP; pmp23::Δpmp23; Str^R*	This work
Plasmids		
pCM83	• [*bgaA::P$_{Zn}$-sfgfp]*<br>Encoding sfGfp under the control of the PZn promoter, allows chromosomal integration at the bgaA locus; AmpR, TetR<br>• Was generated in a two-way ligation with an AgeI-SpeI DNA fragment encoding the *sfgfp* gene and pCM38 [bgaA::PZn-gfp+] (Jacq et al., 2015) cut with AgeI and SpeI.	This work
pMJ1	• [*bgaA::P$_{Zn}$-sfgfp-pmp23]*<br>Encoding sfGfp-Pmp23 under the control of the PZn promoter, allows chromosomal integration at the bgaA locus; AmpR, TetR<br>• Was generated in a two-way ligation with an SpeI-NotI PCR product encoding the pmp23 gene (oligonucleotide primers FORoMJ3 and REVoMJ4 on genomic DNA as template) and pCM83 [bgaA::PZn-sfgfp] cut with SpeI and NotI.	This work
pLB51	• [*bgaA::P$_{Zn}$-sfgfp-pmp23(E61Q)]*<br>Encoding sfGfp-Pmp23(E61Q) under the control of the PZn promoter, allows chromosomal integration at the bgaA locus; AmpR, TetR<br>• Was generated by a site directed mutagenesis with oligonucleotide primer FORoMJ58 and REVoMJ59 from plasmid pMJ1.	This work
pLB52	• [*bgaA::P$_{Zn}$-sfgfp-pmp23(D68N)]*	This work

	Encoding sfGfp-Pmp23(D68N) under the control of the PZn promoter, allows chromosomal integration at the bgaA locus; AmpR, TetR<br>• Was generated by a site directed mutagenesis with oligonucleotide primer FORoMJ147 and REVoMJ148 from plasmid pMJ1.	
pMJ2	• [*bgaA::P*]<br>Encoding 3xflag-Pmp23 under the control of the PZn promoter, allows chromosomal integration at the bgaA locus; AmpR, TetR<br>• Was generated in a two-way ligation with an AgeI-SpeI product encoding the 3x flag epitope (annealing from oligonucleotide FORoMJ1 and REVoMJ2) and pMJ1 [bgaA:: PZn-sfgfp-pmp23] cut with AgeI and SpeI.	This work
pMJ18	• *[pGex4T1-gst-pmp23]*<br>Encoding a fusion protein between the GST and full-length pmp23, Amp^R	8
pGex-PBP2x	• *[pGex4T1-gst-pbp2x]*<br>Encoding a fusion protein between the GST and the extracellular domain of PBP2x (G49-D750), Amp^R	9
pT7-His7-mapZextra2	• *[pT7-His_7-mapZextra2]*<br>Encoding a fusion protein between a polyhistidine tag and the extracellular domain of MapZ (Q182-Y464), Amp^R	6

Table S2. Oligonucleotide primers used in this study

Primer	Sequence	Used for
FORoMJ1	atgaaacatcttaccggtgattataaagatgatgatgataaagattataaagatgatgatgataaagattataaagatgatgatgataaaggttccgctggctccgctgctggttctggcactagttttaaacgaattc	construction of pMJ3
REVoMJ2	gaattcgtttaaaactagtgccagaaccagcagcggagccagcggaaccttatcatcatcatctttataatctttatcatcatcatctttataatctttatcatcatcatctttataatcaccggtaagatgtttcat	construction of pMJ3
FORoMJ3	cgcactagttttaaacgaattcgaagagtg	construction of pMJ1
REVoMJ4	gcggcggccgcttagccagatgttgaaaagagagtgaa	construction of pMJ1
FORoMJ58	gcttgctatgatttatactcaaacaaaaggaaaagaaggcg	E61Q site directed mutagenesis
REVoMJ59	cgccttcttttccttttgtttgagtataaatcatagcaagc	E61Q site directed mutagenesis
FORoMJ147	caaaaggaaaagaaggcaatgttatgcagtctagtgag	D68N site directed mutagenesis
REVoMJ148	ctcactagactgcataacattgccttcttttccttttg	D68N site directed mutagenesis
FOROMJ25	gcttacatgtccttgattgagccagg	amplification upstream of the *pmp23* gene
REVoMJ22	gcgccttatccgaatcaaag	amplification downstream of the *pmp23* gene
FORoMJ23	taaacgaattcgataaggctaaagagtgcttgtactag	STOP codon insertion in the *pmp23* gene
REVoMJ24	ctagtacaagcactctttagccttatcgaattcgttta	STOP codon insertion in the *pmp23* gene

Underlined: restriction sites or STOP codon

1. Grant, S.G., Jessee, J., Bloom, F.R. & Hanahan D. Differential plasmid rescue from transgenic mouse DNAs into Escherichia coli methylation-restriction mutants. *Proc. Natl. Acad. Sci. U S A*. **87**, 4645-4649 (1990).
2. Studier, F.W. & Moffatt, B.A. Use of bacteriophage T7 RNA polymerase to direct selective high-level expression of cloned genes. *J. Mol. Biol.* **189**, 113-130 (1986).
3. Avery, O.T., Macleod, C.M. & McCarty, M. Studies on the Chemical Nature of the Substance Inducing Transformation of Pneumococcal Types : Induction of Transformation by a Desoxyribonucleic Acid Fraction Isolated from Pneumococcus Type Iii. *J. Exp. Med.* **79**, 137-158 (1944).
4. Lefevre, J.C., Claverys, J.P. & Sicard, A.M. Donor deoxyribonucleic acid length and marker effect in pneumococcal transformation. *J. Bacteriol*. **138**, 80-86 (1979).
5. Fleurie, A. *et al*. Interplay of the Serine/Threonine-Kinase StkP and the Paralogs DivIVA and GpsB in Pneumococcal Cell Elongation and Division. *PLOS Genet*. 10:e1004275 (2014).

6. Fleurie, A. *et al*. MapZ marks the division sites and positions FtsZ rings in *Streptococcus pneumoniae*. Nature **516**, 259-262 (2014).

7. Fleurie, A. *et* al. Mutational dissection of the S/T-kinase StkP reveals crucial roles in cell division of *Streptococcus pneumoniae*. *Mol. Microbiol*. **83**, 746–758 (2012).

8. Pagliero, E. *et al*. The Inactivation of a New Peptidoglycan Hydrolase Pmp23 Leads to Abnormal Septum Formation in *Streptococcus pneumoniae*. *Open Microbiol. J.* **2**, 107-114 (2008).

9. Pares, S., Mouz, N., Pétillot, Y., Hakenbeck, R. & Dideberg, O. X-ray structure of *Streptococcus pneumoniae* PBP2x, a primary penicillin target enzyme. Nat. Struct. Biol. **3**, 284-289 (1996).
